# Supplementary material for: An information theoretic approach to detecting spatially varying genes
Source: Cell Rep Methods. 2023 Jun 16;3(6):100507. doi: 10.1016/j.crmeth.2023.100507 (PMC10326450; doi:10.1016/j.crmeth.2023.100507)
Supplement: Document S1. Figures S1–S5 [file mmc1.pdf]

Cell Reports Methods, Volume *3*

Supplemental information

An information theoretic approach

to detecting spatially varying genes

Daniel C. Jones, Patrick Danaher, Youngmi Kim, Joseph M. Beechem, Raphael Gottardo, and Evan W. Newell

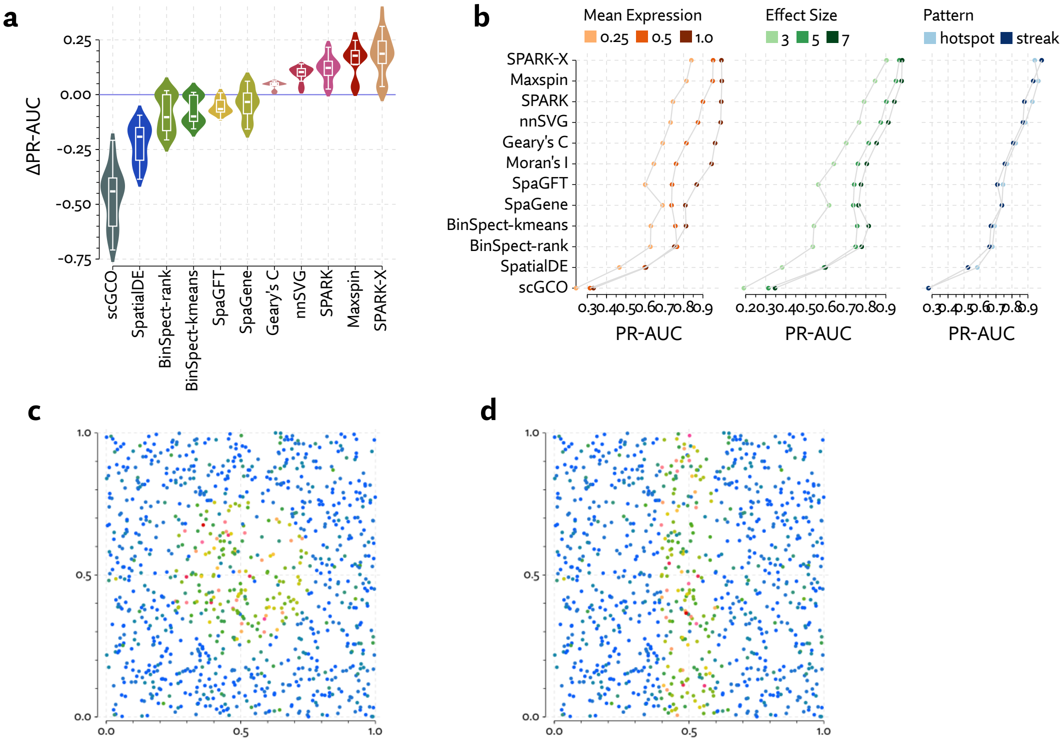

Figure S1: **An alternative simulation benchmark, related to Figure 2.** A simulation consisting uniformly distributed cells with a circle ("hotspot") or vertical band ("streak") of perturbed expression. **a** Aggregate performance is measured by computing the area under the precision-recall curve and subtracting the value obtained by Moran's I. **b** Performance is further broken down by simulation parameters.

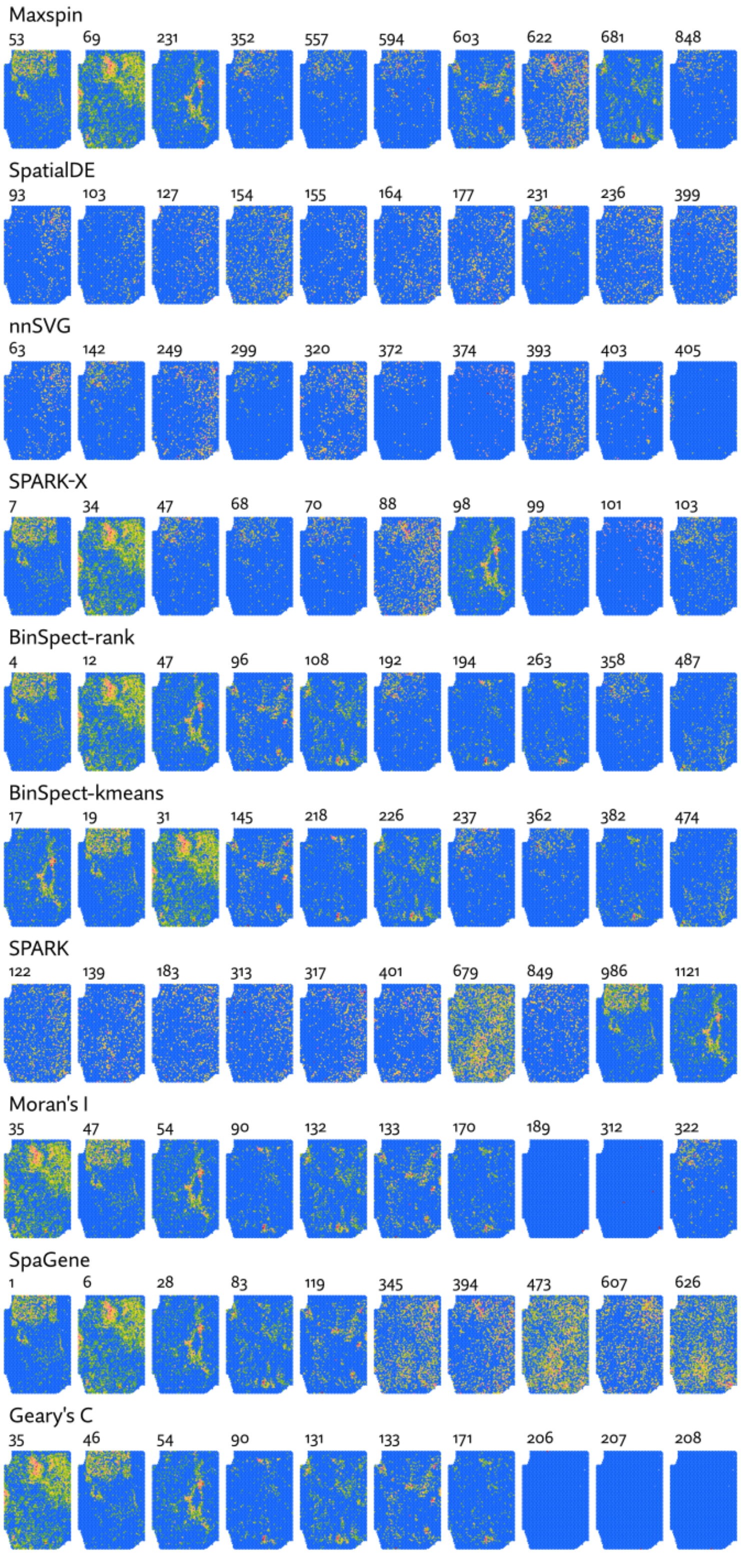

Figure S2: **A comparison of the top-10 highest ranking false-positives across tested methods, related to Figure 3**. Top ranking genes for each method were taken from sample 151509 from the Visium dataset. Listed above each example is it's rank. Many of the same examples appear across methods, but tend be ranked lower by Maxspin.

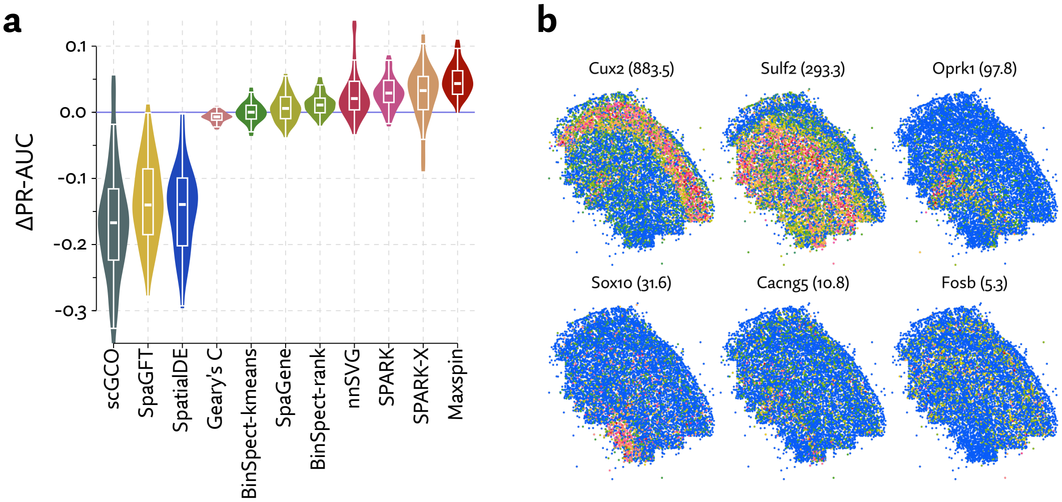

Figure S3: **Additional MERFISH benchmarking, related to Figure 3.** Mouse primary motor cortex MERFISH data was taken from Zhang, et al.[1]. The data consists of panel of 252 genes measured in 12 experiments, each consisting of a number of coronal slices (64 in total), with a total of 280,186 cells. We annotated cortical layers by reproducing the procedure described by the authors, discarding one of the experiments where we could not reliably annotate the L6b layer. (a) results are shown for each applicable method, and (b) examples of SVGs with their accompanying information scores.

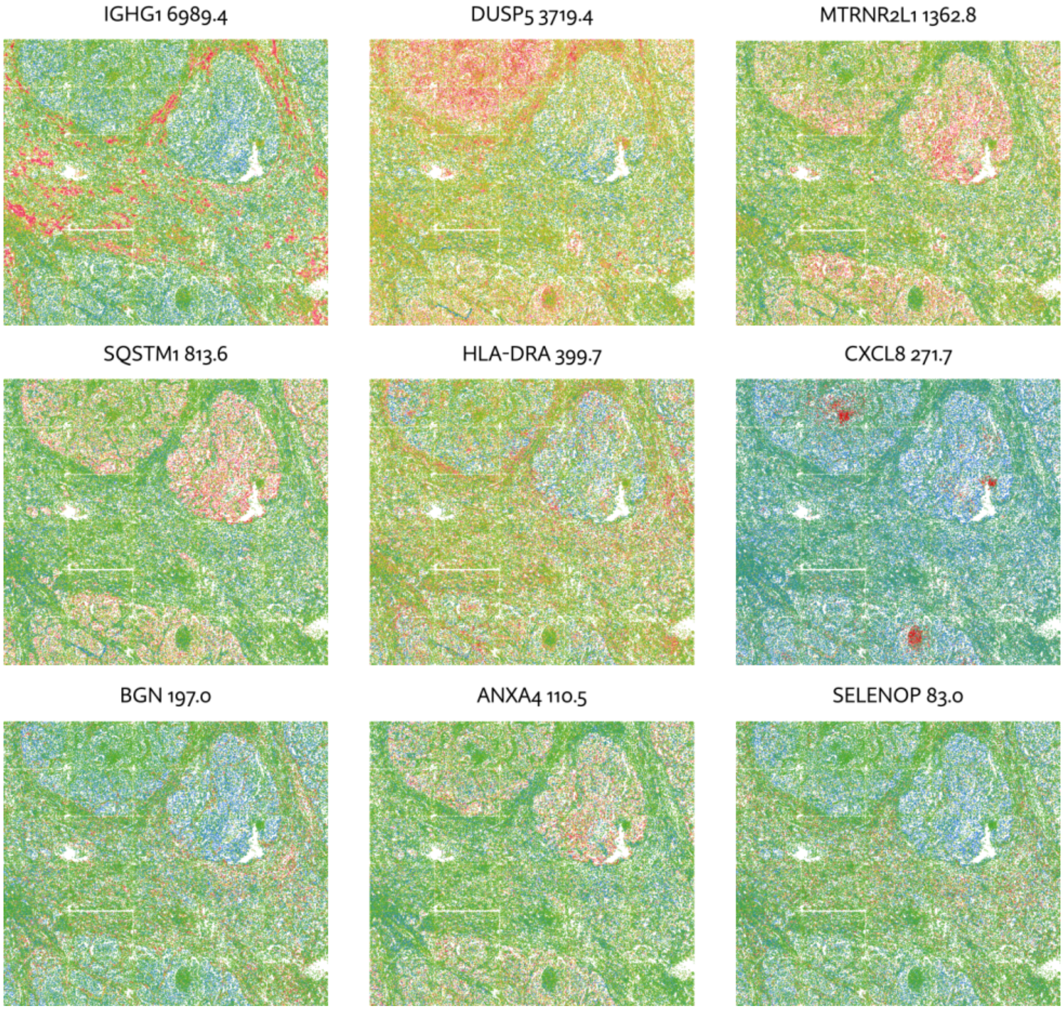

Figure S4: **Examples of genes with corresponding spatial information scores from the renal cell carcinoma CosMx data, related to Figure 4** Genes were selected to show a range of spatial information scores.

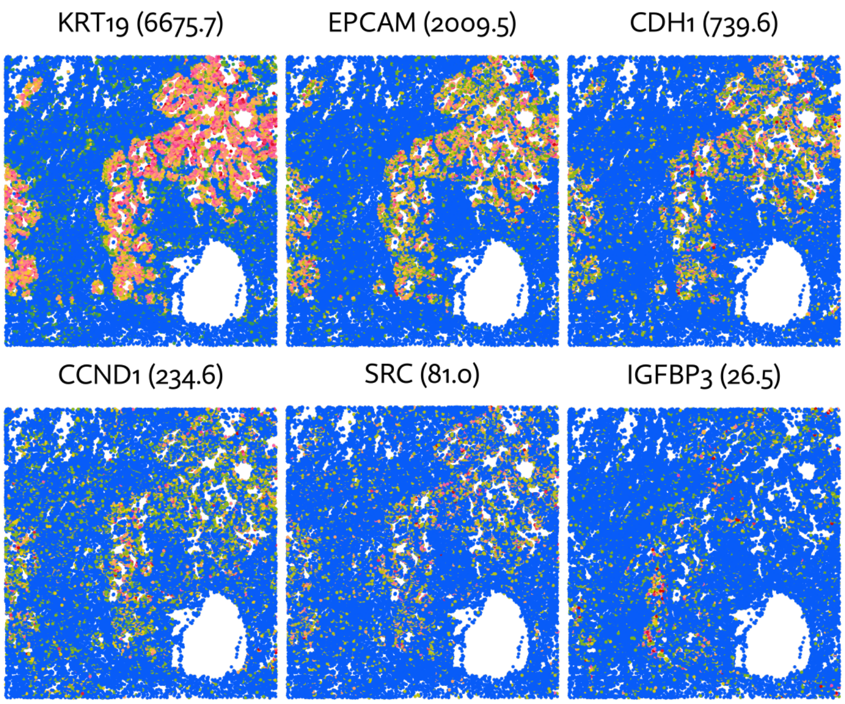

Figure S5: **Examples of genes with corresponding spatial information score, related to Figure 3** Spatial information is seen to strongly correspond to the effect size and the number of cells involved. The highest scoring genes here show elevated expression in the tumor interior.

References

1. Zhang, M., Eichhorn, S. W., Zingg, B., Yao, Z., Cotter, K., Zeng, H., Dong, H., & Zhuang, X. (2021). Spatially resolved cell atlas of the mouse primary motor cortex by MERFISH. *Nature*, *598*, 137–143.
