# Supplementary material for: An information theoretic approach to detecting spatially varying genes
Source: Cell Rep Methods. 2023 Jun 16;3(6):100507. doi: 10.1016/j.crmeth.2023.100507 (PMC10326450; doi:10.1016/j.crmeth.2023.100507)
Supplement: Document S2. Article plus supplemental information [file mmc2.pdf]

Cell Reports
Methods

Article

An information theoretic approach to detecting spatially varying genes

Graphical abstract

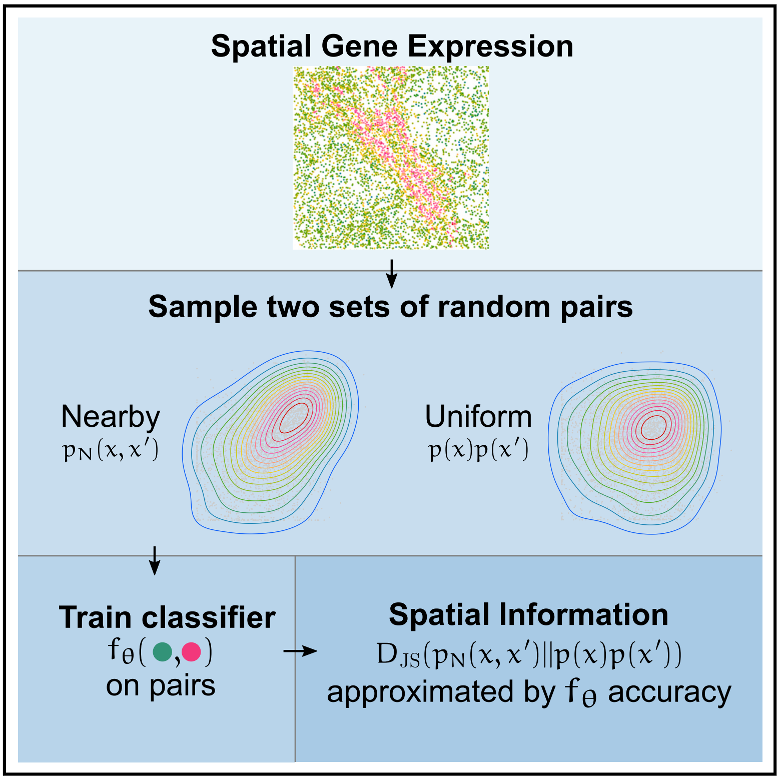

Highlights

- Spatial organization can be formalized in information theoretic terms
- Modern methods from deep learning enable efficient estimation of spatial information
- Spatial information outperforms other methods at identifying spatially varying genes
- Patterns of expression in renal cell carcinoma are revealed using spatial information

Authors

Daniel C. Jones, Patrick Danaher, Youngmi Kim, Joseph M. Beechem, Raphael Gottardo, Evan W. Newell

Correspondence

djones3@fredhutch.org (D.C.J.),
enewell@fredhutch.org (E.W.N.),
raphael.gottardo@chuv.ch (R.G.)

In brief

Quantifying the degree of spatial organization in a biological signal is a key building block of spatial transcriptomics analysis. Jones et al. conceptualize this in information theoretic terms, providing an interpretable and accurate tool to reveal spatial organization.

Jones et al., 2023, Cell Reports Methods *3*, 100507
June 26, 2023 © 2023 The Authors.
https://doi.org/10.1016/j.crmeth.2023.100507

CellPress

Cell Reports Methods

CellPress
OPEN ACCESS

Article

An information theoretic approach to detecting spatially varying genes

Daniel C. Jones,[1,7,*] Patrick Danaher,[2] Youngmi Kim,[2] Joseph M. Beechem,[2] Raphael Gottardo,[1,3,4,5,6,*] and Evan W. Newell[1,6,*]
[1]Fred Hutchinson Cancer Center, Seattle, WA, USA
[2]NanoString Technologies, Inc., Seattle, WA, USA
[3]Biomedical Data Science Center, Lausanne University Hospital, University of Lausanne, Lausanne, Switzerland
[4]Swiss Institute of Bioinformatics, Lausanne, Switzerland
[5]Ludwig Institute for Cancer Research, Lausanne Branch, Lausanne, Switzerland
[6]These authors contributed equally
[7]Lead contact
*Correspondence: djones3@fredhutch.org (D.C.J.), enewell@fredhutch.org (E.W.N.), raphael.gottardo@chuv.ch (R.G.)
https://doi.org/10.1016/j.crmeth.2023.100507

MOTIVATION Identifying genes with spatially coherent expression patterns is a key task in spatial transcriptomics analysis. Previously proposed methods have various shortcomings, often failing to adequately conceptualize the problem, making overly strong distributional assumptions, struggling to scale to the rapidly increasing scale of the data, or failing to demonstrate any improvement over classical spatial statistics methods.

SUMMARY

A key step in spatial transcriptomics is identifying genes with spatially varying expression patterns. We adopt an information theoretic perspective to this problem by equating the degree of spatial coherence with the Jensen-Shannon divergence between pairs of nearby cells and pairs of distant cells. To avoid the notoriously difficult problem of estimating information theoretic divergences, we use modern approximation techniques to implement a computationally efficient algorithm designed to scale with *in situ* spatial transcriptomics technologies. In addition to being highly scalable, we show that our method, which we call maximization of spatial information (Maxspin), improves accuracy across several spatial transcriptomics platforms and a variety of simulations when compared with a variety of state-of-the-art methods. To further demonstrate the method, we generated *in situ* spatial transcriptomics data in a renal cell carcinoma sample using the CosMx Spatial Molecular Imager and used Maxspin to reveal novel spatial patterns of tumor cell gene expression.

INTRODUCTION

The last several years have seen a slew of advancements in spatially resolved transcriptomics,[1,2] proteomics,[3] and genomics.[4] These methods create an opportunity to develop a deep understanding of the spatial organization of tissues at the cellular level. When the expression of a large number of genes is measured, a key consideration is identifying which genes show signs of structured, non-random spatial organization. Analogous to identifying differentially expressed genes, these methods aim to identify spatially varying genes (SVGs).

Similar questions have been examined in geostatistics, ecology, and demography for decades, so naturally, existing spatial statistics methods have been put to use. Traditional measures of spatial auto-correlation, the most common of which are Moran's I and Geary's C,[5] have numerous general purpose implementations but have also been included in spatial expression analysis toolkits like Squidpy[6] and MERINGUE.[7]

A number of methods have built more elaborate, special-purpose models based on Gaussian processes with covariance functions applied over spatial coordinates ("kriging" in geostatistics parlance), which have long been a standard tool in probabilistic modeling of spatial data. SpatialDE[8] tests for SVGs by separately fitting a Gaussian process model and a regression model excluding spatial covariance and comparing the two using a χ^2 test. SPARK[9] elaborates on this primarily by using Poisson likelihood, which is a more natural model of count data, and by testing models with ten different covariance functions to account for various potential patterns of spatial coherent expression. GPcounts[10] models counts using a negative binomial distribution and tests for spatial coherence using a likelihood ratio test

© 2023 The Authors. This is an open access article under the CC BY license (http://creativecommons.org/licenses/by/4.0/).

on the marginal likelihoods of models with and without the prior Gaussian process.

Gaussian process models have many appealing properties, but they also have one major shortcoming: exact inference is $O(n^3)$, where here n is the number of cells or spots. This quickly becomes problematic once n is larger than a few thousand. Given the recent rapid progress in spatial assays, cubic time inference marks a method for rapid obsoletion. A recent method, nnSVG,[11] overcomes this problem by leveraging a modern approximate inference scheme for Gaussian process models. SPARK-X[12] instead bypasses the issue by using an entirely different model than SPARK, a null-hypothesis test on the Frobenious inner product of the spatial coordinate covariance matrix and one computed over a gene's expression. When these are highly non-independent, the inner product test statistic will be large, yielding smaller p values. To consider various patterns of spatial coherence, it runs this test on different transformations of the coordinates.

Apart from the Gaussian process methods, trendsceek[13] uses a mark-segregation test, which tests the statistical independence of the distance between two cells and a gene's expression in both. If conditioning on the distance does not alter the distribution over expression pairs, there is no detectable spatial coherence. Independence is tested using summary statistics, and p values are computed by shuffling expression values across spatial locations to compute an empirical null distribution. Sepal[14] constructs a model simulating continuous time diffusion of expression across the spatial domain and measures spatial coherence by the time required for the process to reach a homogeneous state. SpaGFT[15] uses the graph Fourier transform to decompose an expression signal on a neighborhood graph into a frequency domain, letting it test for significant lower frequency variations. Finally, scGCO[16] bins a gene's expression using a Gaussian mixture model, clusters cells using a hidden Markov random field on the spatial neighborhood graph, and then tests for overrepresentation of expression bins within cell clusters. Other methods based on first clustering cells, such as SpaGCN[17] and STAMarker,[18] are able to detect SVGs but only with respect to specific domains and so are not directly comparable.

Giotto,[19] in addition to interfacing with SpatialDE, trendsceek, and SPARK, implements its own SVG test called binary spatial extraction (BinSpect), which binarizes expression values using either K-means clustering (BinSpect-*k*means) or threshold ranking (BinSpect-rank) and runs a Fisher's exact test using a contingency table of values from neighboring cells. SpaGene[20] also heuristically binarizes expression data but instead considers the degree distribution for a subgraph of the k nearest neighbor graph consisting of just high expression cells, operating under the principle that if high expression cells collocate, we would expect an increase in higher degree nodes in this graph when compared with a shuffled graph.

Existing methods often make either strong distribution assumptions, discard information through binarization, or are highly tuned in an ad hoc manner to specific types of patterns. In the work presented here, we develop an entirely new approach to measuring spatial coherence, adopting an information theoretic perspective and building on recent advancements in approximating mutual information. Like trendsceek, our goal is to quantify the degree of statistical dependence between a pair of expression values and their spatial vicinity, but rather than running null hypothesis tests on summary statistics, we take a more direct route by estimating the Jensen-Shannon (JS) divergence between the two, computing a score analogous to mutual information. Spatial coherence can then be neatly defined as how much information gene expression values in the same neighborhood share. In a spatially incoherent setting, this divergence is low; nearby expression values share no more information than randomly selected values. When expression is organized spatially, expression pairs become increasingly predictable based on whether they are nearby or not, and this divergence will be high.

Information theoretic divergences are appealing on theoretical grounds, but their application is often fraught. Computing mutual information directly necessitates a model of the joint probability and, in the case of continuous domains, computing a multidimensional integral. Recently, mutual information has gained renewed interest in the context of deep learning.[21] Belghazi et al.[22] showed, in a method they call mutual information neural estimation (MINE), how mutual information between to variables X, Y can be estimated by training a neural network classifier to distinguish pairs of observations (*de facto* draws from the joint distribution $P(X,Y)$) from pairs drawn from the marginal distributions $P(X)$,$P(Y)$, which can be formed by shuffling observations. Intuitively, the better the neural network is able to distinguish observed pairs from shuffled pairs, the higher the mutual information. This intuition is formalized by showing that the objective function used to train the neural network forms a lower bound on the mutual information.

In our method, Maxspin (a portmanteau of "maximization of spatial information"), we adopt an approach similar to MINE to compute lower bounds on the JS divergence between pairs of nearby and pairs of distant gene expression values. On a gene-wise basis, we optimize a simple classifier to distinguish pairs of expression value pairs chosen uniformly at random across spatial locations from pairs chosen from nearby locations according to a short random walk on the spatial neighborhood graph (Figure 1A). Spatial coherence is scored as the degree to which a classifier can recognize uniformly sampled pairs from spatially proximate pairs, with an objective function that is a lower bound on the JS divergence between the two distributions, representing a measure of spatial auto-information, which we will refer to as simply "spatial information" here. This principle can be trivially generalized to consider the spatial information between pairs of genes, quantifying the degree of spatial coexpression. Maxspin is available under an open source licence at https://github.com/dcjones/maxspin.

RESULTS

We used two strategies to test the performance of Maxspin compared with existing methods of detecting SVGs. First, we developed a simulation framework in which a subset of genes are simulated as having subtle spatial organization. Second, we used three publicly available datasets generated using different spatial transcriptomics technologies: 10× Visium, NanoString CosMx Spatial Molecular Imager (SMI), and Vizgen

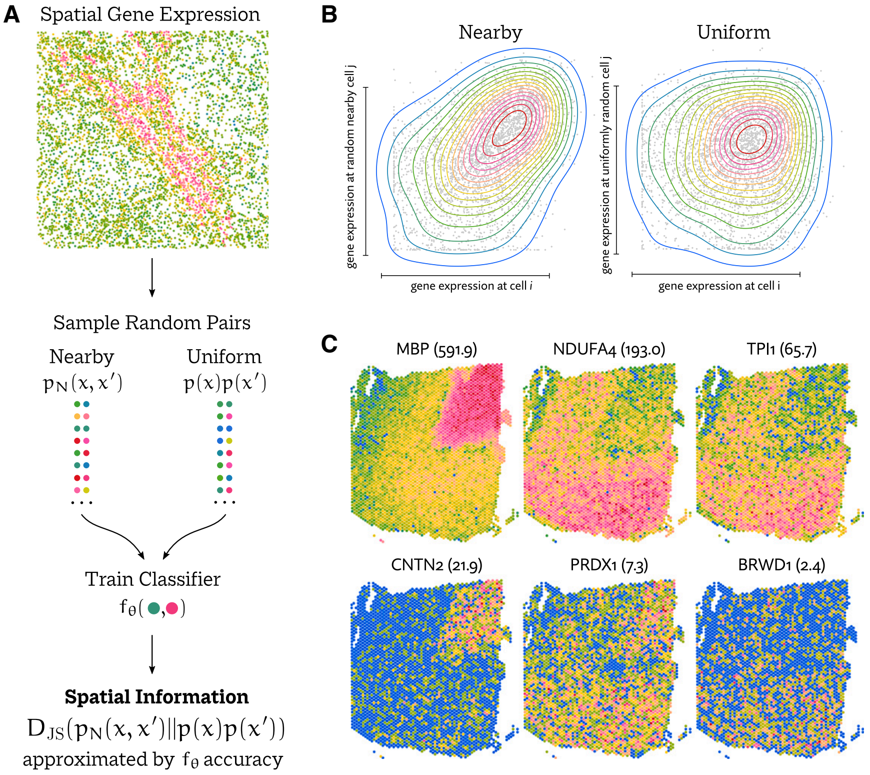

Figure 1. Overview of the Maxspin algorithm for detecting spatially varying genes

(A) The maximization of spatial information (Maxspin) algorithm proposed here works by training a simple classifier to distinguish between expression pairs sampled from nearby locations and those chosen uniformly at random.

(B) If a gene displays spatial organization, there is a distributional shift between the two sets of sampled cell pairs. The classifier accuracy acts as an approximation of the Kullback-Leibler divergence between these distributions, which can be interpreted as spatial information.

(C) The spatial information score computed effectively quantifies the degree of spatial coherence for each gene evaluated, here shown in a Visium mouse brain dataset.

MERFISH. The simulation has the advantage of known ground truth.

For the real data, as a proxy for ground truth, we annotated spatially varying clusters of cells or spots and called differential expression between these using pairwise Wilcoxon signed-rank tests and a Benjamini-Hochberg q value cutoff of 0.01. This list no doubt omits some SVGs with spatial expression patterns not conforming to the annotated regions, but we expect it to contain the large majority of the most obvious examples, and omissions should equally disadvantage each method and not *a priori* bias the benchmark toward any particular method.

Because of the wide variety of settings, we adopt Moran's I as a baseline method and consider the degree to which a method improves on (or falls below) its performance, as measured by the area under the precision-recall curve (PR-AUC). Moran's I is simple, easy to interpret and implement, and has been a mainstay of spatial statistics for 70 years. Thus, it is a meaningful hurdle that any specialty method for detecting SVGs should aim to surpass.

GPcounts was excluded from these benchmarks. Though it has a test of spatial coherence, it was designed for very small numbers of cells and fails to scale to any of the datasets evaluated here. We were also unable to run trendsceek as the software is unmaintained and has been rendered unusable by library incompatibilities.

Simulations

To explore a variety of scenarios with a known ground truth, we simulated spatial expression data. The simulation assumes some number of discrete cell types and genes. Random expression values were drawn from negative binomial distributions. For 7,000 of the 8,000 simulated genes, this distribution is fixed across cell type, and for the remaining 1,000 it is perturbed, representing a ground-truth subset of SVGs. To simulate the spatial arrangement of cells, we developed a cellular Potts model[23] in which cells migrate and deform according to an actin-inspired mechanism.[24] Cells adhere to one another with greater or lesser strength according to a random cell type affinity matrix.

To consider conditions of more or less spatial coherence, we initialized cell positions into highly ordered arrangements by assigning cell type according to sine waves varying across the spatial dimensions, producing homogeneous clumps of cells. Simulation parameters were then chosen so that cell arrangements slowly grew more disordered with more iterations while still displaying some patterning due to varying cell type adhesions. Thus, running the simulation for a larger number of iterations produces an arrangement of cells with spatial patterns that are subtler but still present. We ran a variety of simulations with 2,000 and 10,000 cells. Because SPARK and SpatialDE are both Gaussian process methods, the former with $O(n^3)$ inference and the latter limited by GPU memory, they were unable to scale to the $n = 10,000$ simulations.

Maxspin was found to outperform other methods by a large margin in these simulations (Figures 2A and 2B). We varied parameters of the simulation, including setting the negative binomial dispersion parameter r between 3 and 6, the number of cell types between 2, 4, and 8, and the number of simulation iterations between 10, 100, and 1,000. These results hold up across these different parameterizations (Figure 2C). SPARK and nnSVG compete for second, but SPARK fails to scale to 10,000 cells, and SPARK-X shows wildly variable performance. Geary's C slightly improves on Moran's I, perhaps due to more subtle local variation, while all other methods consistently underperform Moran's I.

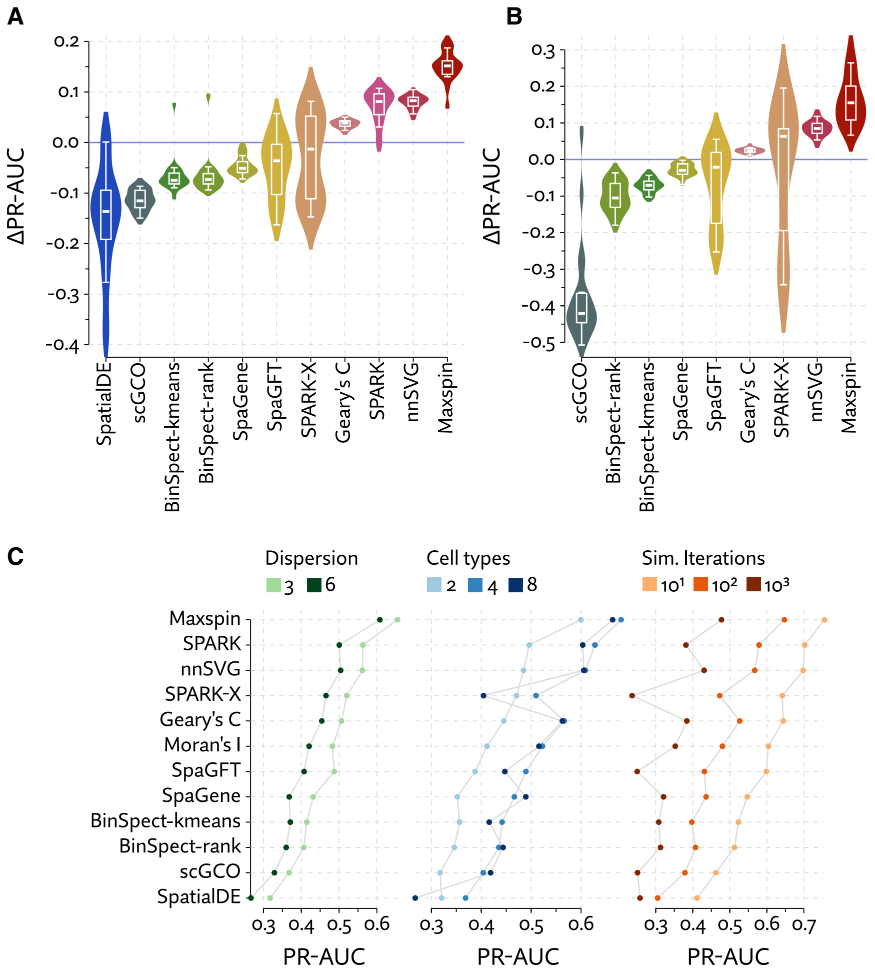

Figure 2. Evaluation of the performance of methods to detect spatially varying genes using simulated data

(A and B) Spatial transcriptomics data were simulated using a cellular Potts model for (A) 2,000 and (B) 10,000 cells, with expression drawn from a negative binomial distribution that is perturbed across cell types for the ground-truth spatially varying genes. Methods were benchmarked by computing the area under their precision-recall curves and subtracting the value achieved by Moran's I.

(C) Performance is further broken down by various parameterizations of the simulation. Across the board, performance increases with lower dispersion and fewer simulation iterations (resulting in simpler spatial distributions), with the number of cell types playing a more ambiguous role.

We additionally adapted simulations from Zhu et al.[12] consisting of cells distributed uniformly at random across a rectangle, with a subset of SVGs that have modulated expression either in a single vertical band region ("streak") or a circular region ("hotspot"), examples of which are shown in Figures S1C and S1D. We generated a number of simulations by varying parameters controlling mean expression, overdispersion, and effect size. The cosine and Gaussian coordinate transformations used by SPARK-X are well tuned to detect these particular simple spatial patterns, yet Maxspin achieves largely equivalent performance without any specialty spatial kernels (Figures S1A and S1B). As with the cellular Potts simulation, SPARK and nnSVG compete for second place, Geary's C slightly surpasses Moran's I, and all other methods underperform Moran's I. We might expect SpatialDE to perform similarly to the other Gaussian process methods, but it once again assigns 0 p values to many examples, erasing any meaningful ordering.

Because most biological instances of spatially coherent expression, especially at the cellular level, are so much more complex, we believe that these streak and hotspot simulations are less realistic than our cellular Potts model, yet they do offer an important alternative perspective. SPARK-X does well here, but considerably worse on the more sophisticated cellular Potts model, with accuracy collapsing in some cases of subtle spatial organization, while Maxspin very consistently performs well across all simulations.

Visium

We used human prefrontal cortex data from Maynard et al.,[25] generated on the 10× Visium platform. The expression of 21,151 genes was profiled in 12 samples. Visium slides consist of a grid of 4,992 spots laid out in a hexagonal pattern. In this dataset, between 3,460 and 4,789 spots were covered by the tissue samples. Compared with single-cell methods, Visium spots are typically much more deeply sequenced but are lower resolution, containing several cells in most cases. To determine a ground-truth set of SVGs, we used the authors' annotation of cortical layers, calling differential expression between all pairs of adjacent layers, resulting in 7,775 SVGs on average.

Every method considered was able to scale to the Visium data by virtue of having a strict maximum number of spots. In future generations of the technology with more spots, this will become more problematic for SPARK and SpatialDE, for which this dataset pushes the limit on tractability. Overall, we demonstrate consistently improved accuracy using Maxspin (Figure 3A). Following Maxspin, the two Gaussian process methods SpatialDE and nnSVG perform well, while SPARK-X was inconsistent, showing high accuracy for some of the 12 samples but greatly diminished for others. SPARK and BinSpect offer only very small improvements over Moran's I, while Geary's C and SpaGene tend to underperform it. SpaGFT, Sepal, and scGCO all performed very poorly with respective median Δ PR-AUC values of −0.52, −0.49, and −0.42. They were excluded from the plot for clarity. Sepal requires data on a regular grid, so we were only able to run it on this benchmark. In Figure 1C, a span of information scores computed by Maxspin is shown for one sample in this dataset.

CellPress
OPEN ACCESS

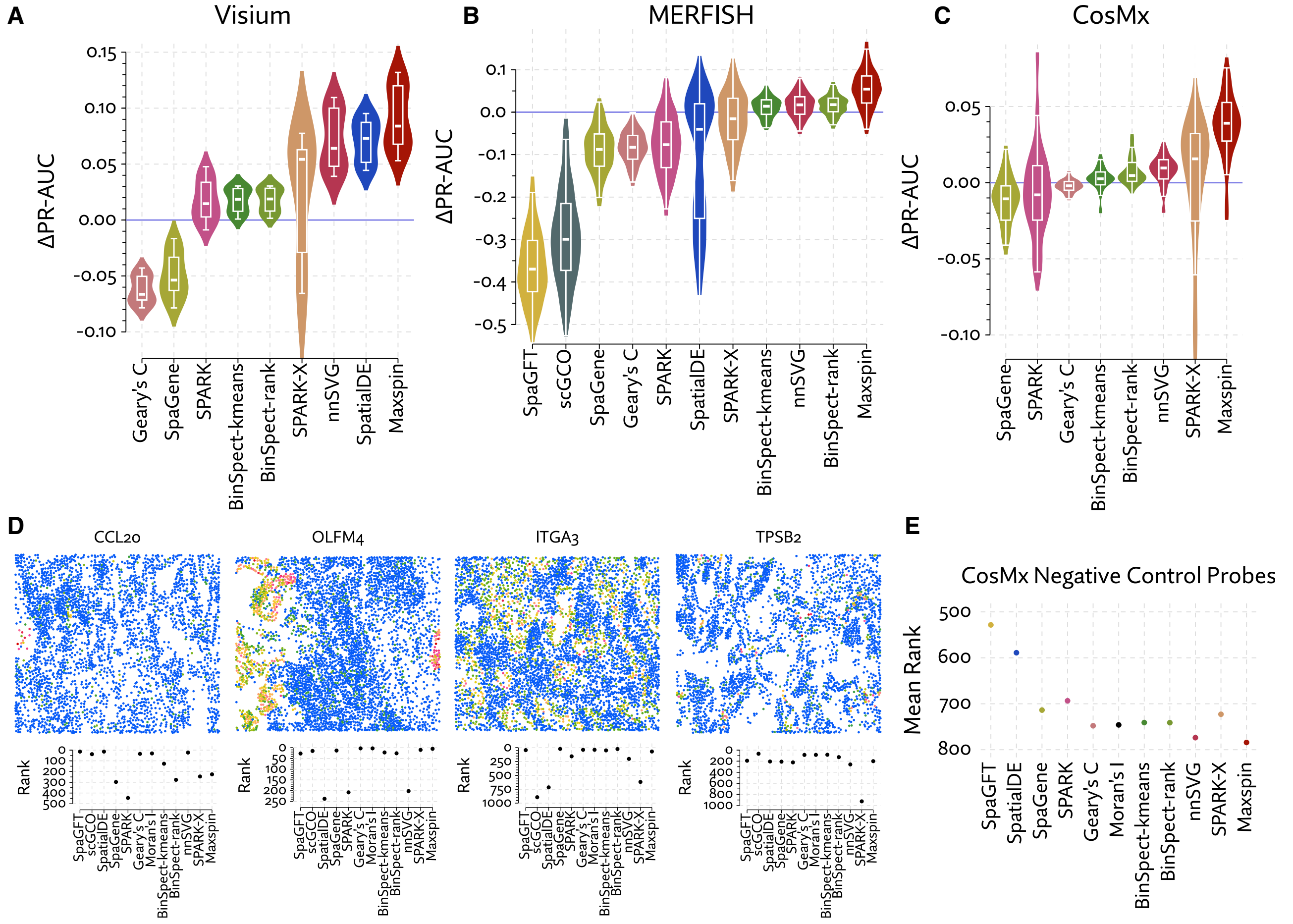

Figure 3. Methods for detecting spatially varying genes were evaluated by calling differential expression between manually annotated regions and treating this list as ground truth

(A–C) The area under the precision-recall curve (PR-AUC) was computed for each test, and the value achieved by Moran's I was subtracted, producing Δ PR-AUC, representing the improvement over this baseline method. Methods were evaluated in multiple datasets generated using (A) Visium, (B) CosMx, and (C) MERFISH. Results for SpatialDE, scGCO, and SpaGFT in the CosMx benchmark were exceedingly low and so were excluded from these plots.

(D) Selected examples of disagreements between methods in the CosMx dataset.

(E) Median ranks (out of 960 genes) of negative control probes in the CosMx dataset, which should not be significantly expressed nor spatially coherent. Not shown is scGCO, with a median rank of 308.

MERFISH

A human lung cancer MERFISH dataset was taken from the Vizgen MERFISH FFPE Human Immuno-oncology dataset,[26] consisting of 500 genes measured across 270,968 cells. With a deliberately selected panel of genes and a large number of cells, many, if not all, genes will display some degree of spatial organization. To construct a benchmark with fewer and harder to detect SVGs, we downsampled counts to 100 per cell and then split the data into regions along a 30 × 30 grid, retaining any region with at least 300 cells, resulting in 508 regions. We jointly clustered cell types across the full data, manually selecting clusters with obvious spatial organization; we then called differential expression between pairs of spatially organized clusters separately on each region. The result is a region-specific lists of SVGs. Each method was then run on individual regions and compared with these lists.

Maxspin has the highest median improvement over Moran's I. Consistent with other benchmarks, nnSVG performs well, SPARK-X and SpatialDE are again seen to be somewhat inconsistent, and other methods underperform the baseline method, Moran's I. SpaGFT and scGCO performed very poorly, with respective median Δ PR-AUC scores of −0.37 and −0.30. These appear to fail in different ways: SpaGFT assigns very low p values to genes with only a small number of counts, while scGCO appears to assign a p value of roughly 0.5 to most genes.

We benchmarked using one additional MERFISH dataset, profiling a mouse primary motor cortex dataset. This also showed improved performance by Maxspin, though to a lesser degree due to the simpler patterns of spatial organization (Figure S3).

CosMx SMI

CosMx SMI is a recently introduced platform from NanoString for spatial profiling of RNA and protein expression. We used CosMx

data from He et al.[27] to evaluate detection of SVGs. Expression of 960 genes was measured across roughly 800,000 cells, across 8 human non-small cell lung cancer samples. We relied on the authors' annotation of tissue microenvironments. We tested individual fields of view for each sample, in part to create a variety of scenarios but also to allow SPARK and SpatialDE to be run on these data, as they ordinarily would struggle to scale to this many cells. Our ground-truth set of SVGs consisted of any gene that varied between any pair of microenvironments, which was on average 419 per field of view.

Maxspin had consistently higher performance that competing methods (Figure 3C). SPARK-X sometimes improved on Moran's I significantly, but as with the Visium data, it is inconsistent, producing poor results on other samples. The remaining methods show performances close to or sometimes below that of Moran's I. As with the MERFISH data, and in stark contrast to the Visium data, SpatialDE showed poor performance, with a median Δ PR-AUC of -0.197. Again, scGCO and SpaGFT also showed very poor performance, with median Δ PR-AUC values of -0.298 and -0.134, respectively.

Figure 3D shows several examples of large disagreements between methods. Often methods will disagree on the relative significance of very small neighborhood of cells with elevated expression (e.g., CCL20), with Maxspin preferring patterns involving more cells. The Gaussian process methods seem to occasionally fail on examples with large unoccupied areas (e.g. OLFM4). SPARK-X produces some unpredictable results, failing to identify fairly obvious examples when they consist of sporadic small regions spread throughout the sample (e.g., ITGA3, TPSB2).

The probe set includes 20 negative control probes, which we expect to be unexpressed. Thus, any non-zero counts for probes represents pure technical noise that should not be detected as spatially varying. Figure 3E shows that while these are typically ranked lowly by all methods, the median rank for Maxspin is the lowest, with nnSVG very nearly tied.

In Figure S5, we show additional examples of spatial information scores computed by Maxspin on small sections of one sample. The highest scoring genes are within tumor regions, which is unsurprising given their specific spatial locality.

Spatially varying expression in renal cell carcinoma

We used Maxspin to investigate patterns of SVG expression in a CosMx dataset of a human renal cell carcinoma (RCC) sample, consisting of 178,410 cells and 993 genes, with an average of 222 counts per cell. The data involved both a large number of cells, which many existing methods fail to scale to, and relatively sparse counts, introducing uncertainty that many methods fail to account for. Maxspin is well suited to this data, as it scales to very large numbers of cells and is able to account for uncertainty under a variety of distributional assumptions.

We first identified cell types using unsupervised clustering of expression data alone. Count data were normalized using Sanity[28] and then clustered using Leiden.[29] This produced 24 clusters, to which we assigned names by comparing average expression across clusters, making use of an existing catalog of human kidney cell types[30] (Figure 4C).

Computing spatial information across all cells will often recapitulate markers for known cell types. For example, the highest scoring gene in this dataset is the immunoglobulin gene IGHG1, revealing populations of B cells that we could have annotated by other means. To reveal more subtle spatial patterns, we instead separately ran Maxspin on each cluster to discover patterns of spatial variation that are less visible to standard clustering methods. Many examples of spatially coherent intra-cluster variations were found in the tumor cell clusters (Figure 4B).

Many of these genes have been studied in the context of RCC. For example, GPX3, VIM, and IGFBP3 are known to be important markers in clear cell RCC,[31,32,33] but much less is known about their patterns of spatial expression within the tumor. DUSP5, which in varying contexts can act either as a tumor suppressor or promoter,[34] is seen to be highly expressed specifically in the upper left tumor region. Curiously, the adjacent tumor region shows distinctly high expression of the MT-RNR2 like-1 pseudogene. MMP7 is upregulated along the tumor-stroma boundary, consistent with its role in degrading extracellular matrix to facilitate tumor invasion.[35]

In non-tumor cell clusters, a number of interesting examples of SVGs also emerge (Figure 4A). Tumor-associated macrophages disproportionately show upregulation of SPP1 and downregulation of CD163, consistent with prior findings.[36,37] Along the tumor-stroma boundary, extracellular matrix genes fibronectin (FN1) and collagen type 4 alpha 1 (COL4A1) are highly expressed in fibroblasts and endothelial cells, respectively. The transcription factor X-box binding protein 1 (XBP1), which is involved in plasma cell differentiation,[38] shows spatially varying clusters of high expression within one of the B cell clusters. Within an epithelial cell cluster, SERPINA1 shows elevated expression surrounding one of the tumor regions but not the others.

A gene's disaggregated information score can be plotted as a saliency map, which provides clear visual explanations for why a gene scores highly. Examples of SVGs with a span of spatial information scores are shown in Figure S4. Not surprisingly, high expression is typically a necessary condition for a high information score, but it is not a sufficient condition, as some high expression genes have relatively low spatial information.

The spatial auto-information score can be trivially generalized to compute the spatial information between a pair of genes. Doing this for all $O(n^2)$ pairs is typically intractable unless n is small, but if we first filter to consider only genes with high auto-information, this becomes a viable way to explore the spatial relationships between genes, which avoids coercing cells into distinct types or clusters where they might not always unambiguously fit.

We chose genes with a spatial information score above 20, resulting in 48 genes, and then computed pairwise information between these genes. Hierarchical clustering of this pairwise information matrix reveals specific relationships of spatial organization among these genes (Figure 4D). Twenty of the genes were broadly tumor associated yet, as indicated by the structure of the dendrogram, show varying patterns of expression within the tumor. Outside of the tumor-associated group, we see some predictable groupings, which we have broadly identified as B cell/immunoglobulin, extracellular matrix, tubule, major histocompatibility complex (MHC), housekeeping, and long non-coding RNA (lncRNA). There is significant variation within these groups, for example the MHC class I gene B2M is in the same tree as four MHC class II genes, but at a much greater distance.

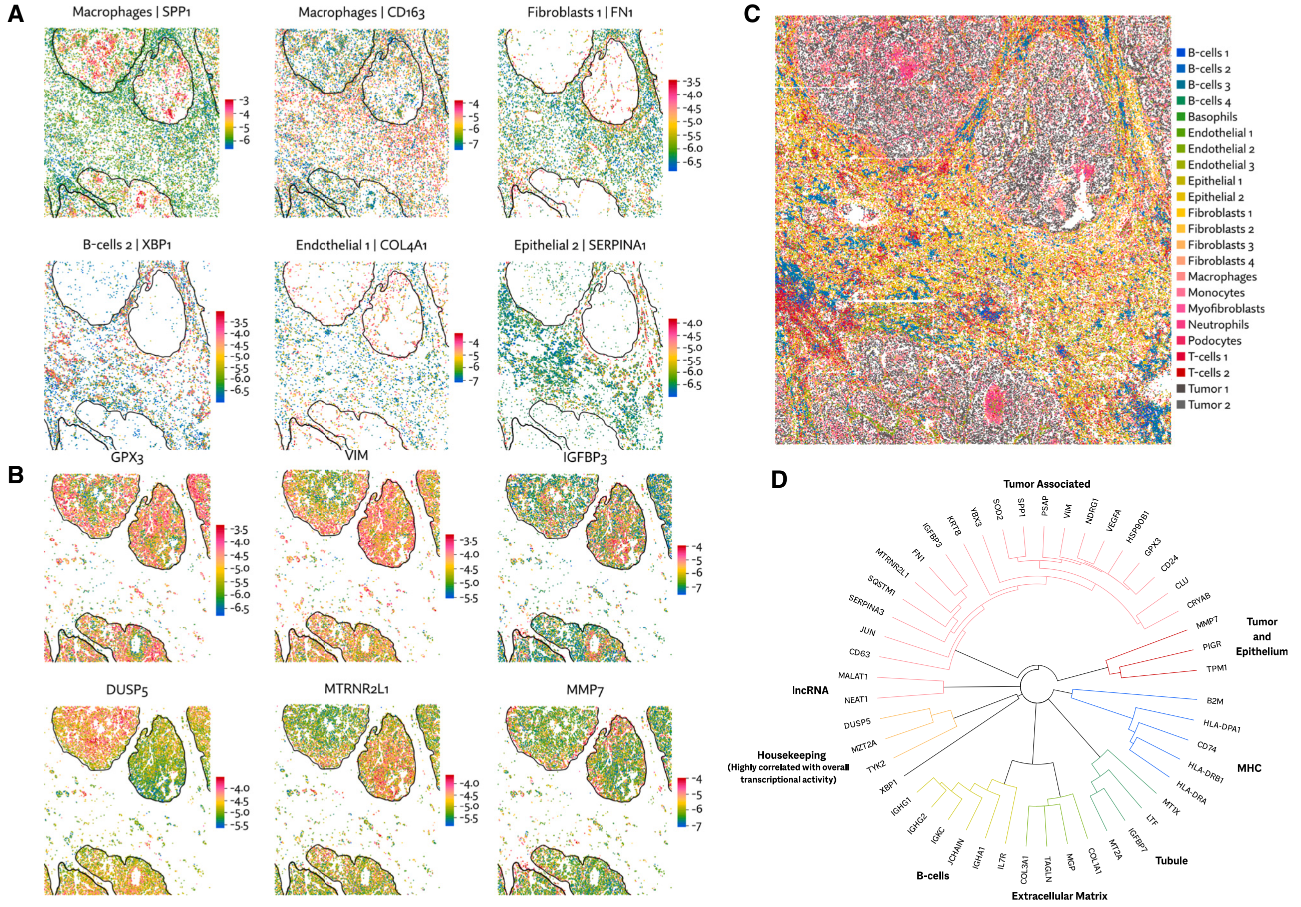

Figure 4. Using Maxspin to analyze the spatial variation of gene expression in a human renal cell carcinoma sample assayed with CosMx

(A) Log expression of spatially varying genes detected in non-tumor cell types. Major tumor regions are indicated by outlines.

(B) Log expression of spatially varying genes detected in tumor cell types.

(C) Classification of cell types using an unsupervised, spatially unaware clustering.

(D) Hierarchical clustering of spatially varying genes using pairwise spatial information.

XBP1 shows a highly distinct pattern of expression, with low spatial information with any other of the genes. Taken together, this gives a rough map of the spatial organization of a subset of genes in this dataset.

Performance

To evaluate computational costs of the methods, we ran them on simulated data with varying numbers of cells. Since these methods test each gene individually, they all scale linearly with the number of genes, and thus we kept the number of genes fixed at 8,000.

The results of this benchmark are shown in Figure 5. SPARK was by far the most computationally expensive method in terms of time and memory. We ran benchmarks for SPARK only out to 5,000 cells, as it became exceedingly time consuming. Most methods scale roughly linearly with the number of cells. The exceptions are the Gaussian process methods SPARK and SpatialDE. The one other Gaussian process method, nnSVG, manages to achieve linear time inference but was still the second slowest method. SpatialDE overtakes nnSVG at 10,000 cells but is faster for fewer cells in part because it runs on a GPU.

To avoid giving Maxspin any opportunity for unfair advantage, we disabled convergence checks when optimizing and let it run for the maximum number of iterations. As a result, the results reported for Maxspin are strictly an upper bound on inference time. Regardless, it significantly outperforms Gaussian process methods, though it cannot match the performance of methods like Moran's I, with simple closed form computations.

SpatialDE and Maxspin differ from the other methods in that they run on GPUs. Though it is possible to run Maxspin without a GPU, it is not designed or optimized to do so. While not advised, running Maxspin without a GPU is possible and is still considerably faster than SPARK. GPU methods also have to work within the constraints of GPU memory, which is typically much more limited than system memory (e.g., 12 GB GPU memory vs. 64 GB system memory in the system we used for these benchmarks). Maxspin is designed to split work up and run in batches, avoiding an issue with memory constraints until spatial transcriptomics technology scales to orders of magnitude larger.

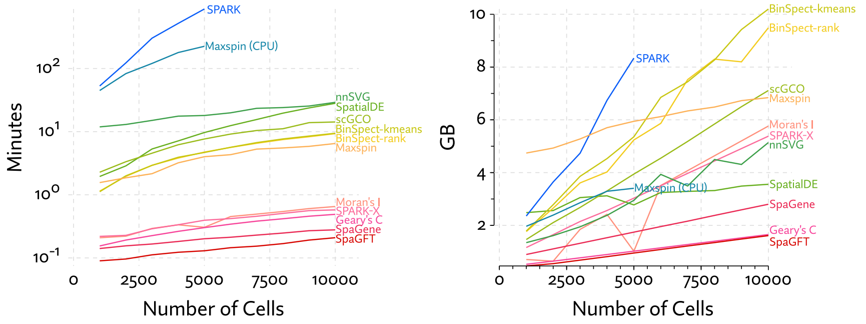

Figure 5. Run time and system memory usage were measured for each method across simulated datasets with varying numbers of cells

Most methods scale linearly with the number of cells, whereas SPARK and SpatialDE have observable different trajectories, as they rely on Gaussian process models with exact inference.

SpatialDE, on the other hand, does rapidly run up against this limitation. Indeed, memory usage is inherently higher in Gaussian process inference, even when approximate inference methods are used.

All benchmarks were performed using Nvidia GeForce 3080 Ti GPU and AMD Ryzen 5950X CPU.

DISCUSSION

The spatial information score computed by Maxspin appears to better capture spatially coherent expression patterns than existing methods across various datasets and simulations. When presenting the results as Δ PR-AUC values, quantifying the improvement over Moran's I, a simple and well-established definition of spatial correlation, we see that specialized modern methods are not always an improvement. Methods aimed at detecting SVGs are sometimes guilty of disregarding decades of work in spatial statistics and fail to demonstrate a consistent advantage.

Taking the benchmarking results together, we see some patterns emerge. Methods that rely on binning or binarization of expression data—SpaGene, scGCO, and BinSpect—clearly discard some useful information and consistently underperform Moran's I. In the case of scGCO, this underperformance is dramatic, typically failing to detect any SVG that is not highly expressed, suggesting that binning is not the only issue. Uniquely among the tested methods, scGCO tries to first cluster cells into spatial domains and then use these domains to inform the test of spatial organization. Other methods such as SpaGCN[17] and STAMarker[18] follow a similar approach, except they detect SVGs specific to each of the identified spatial domains, which is difficult to compare directly with the methods discussed here. Conceivably, if the spatial domains are accurately identified and account for the bulk of spatial variation, this could be a sensitive test. Yet, if spatial variation is not confined to disjoint homogeneous domains and instead varies in a more complex fashion, there is potentially much these methods would miss.

Gaussian process methods—SpatialDE, nnSVG, and SPARK—often perform well. In the case of SpatialDE, it performed very well on the Visium benchmark, and very poorly elsewhere, producing a preponderance of 0 p values, which is a numerical issue that could conceivably be fixed. SpaGFT takes a unique approach by using the graph Fourier transform but generally fares poorly, particularly, it seems, in low-expression examples. SPARK, which uses exact inference, rapidly becomes intractable for large numbers of cells. SpatialDE also fails to scale, though it uses approximate inference. This leaves nnSVG as the clear state of the art in Gaussian process-based tests. However, each Gaussian process model lags behind Maxspin. This is in part due to Maxspin fitting a discriminative model rather than the generative models that the Gaussian process methods involve. Weaker distributional assumptions are involved, making it less prone to influence by small numbers of outlier cells.

SPARK-X is an appealing alternative that is highly efficient and adept at detecting certain spatial patterns. The set of coordinate transformation kernels it uses yields a test that is highly sensitive to, for example, circular regions and bands that span the sample, as demonstrated most distinctly in their simulation, but also in the Visium and an MERFISH brain samples, which have similar bands of distinct cell types. When spatial patterns get more subtle and local, most clearly shown in the cellular Potts simulation, it can entirely fail to detect them. Maxspin excels in this setting and also very nearly matches SPARK-X in sensitivity to the very simple, broad patterns that SPARK-X is specially tuned to.

Maxspin also offers improved explainability compared with more opaque statistical models. Because the information score is a sum across cells, the individual cell-level values can be visualized, showing precisely which cells and regions contribute to a high information score (Figure 6). No other method yet proposed can be disaggregated in this way.

In our analysis of RCC using CosMx SMI, we demonstrated how Maxspin can be used to uncover patterns of expression in a high-dimensional yet relatively sparse dataset by scaling linearly with the number of cells and accounting for uncertainty in expression estimates. Though we largely focused on auto-information, pairwise information is a simple extension that allows genes to be clustered into an atlas of similar and dissimilar spatial patterns. As demonstrated by first clustering into cell types and then computing spatial information, spatial analysis often uncovers patterns of expression that traditional clustering approaches are insensitive to. The clearest indication of this is the several examples of spatially organized expression within the tumor regions. This variation in expression resists explanation by clustering into cell types and is revealed most clearly by gene-level spatial analysis.

The principle presented here, using a discriminative model to maximize a bound on JS divergence, has other potential uses that we have yet to fully explore. An obvious extension is to directly control for possible covariates by estimating a score similar to conditional mutual information. This is straightforwardly achievable by fitting the model twice, once including expression values and the covariate and again with just the covariate, and

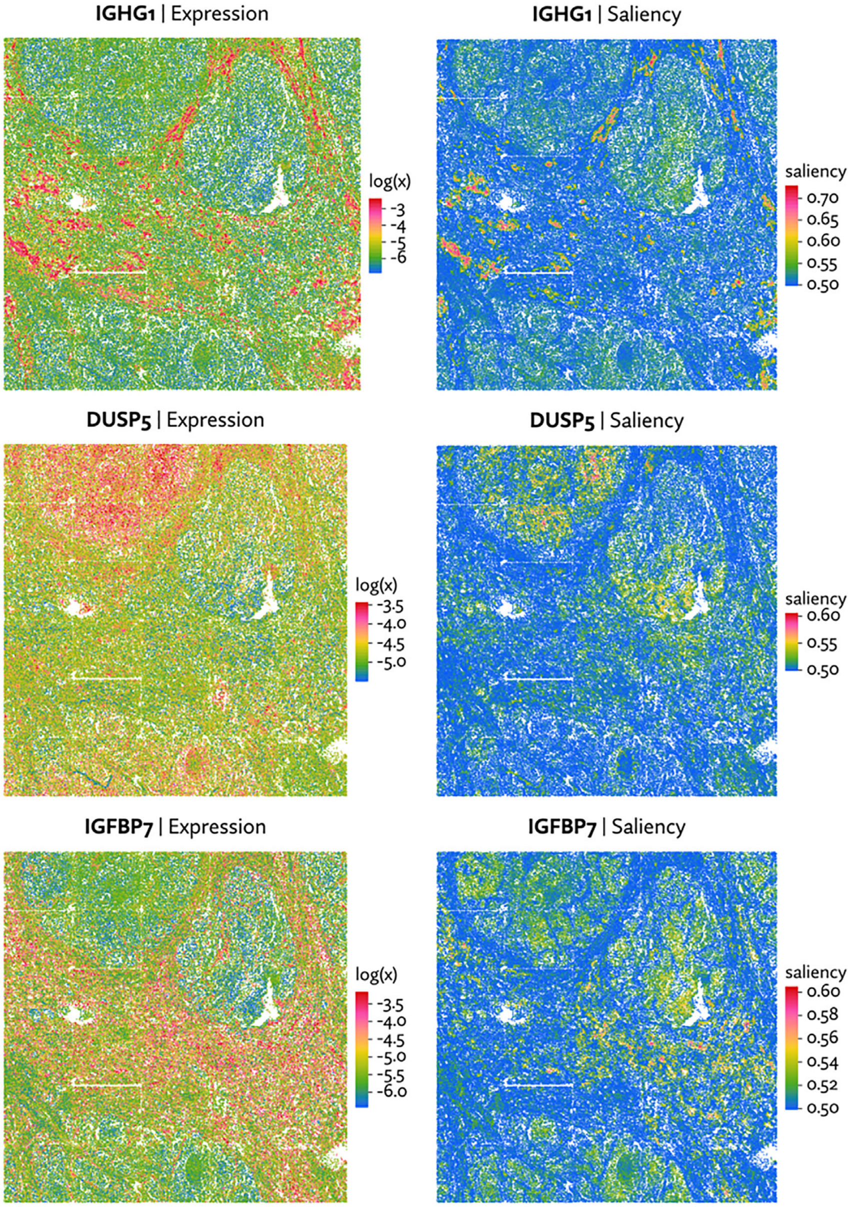

Figure 6. Expression plotted alongside saliency maps, which indicate the cell-level classifier accuracy

High saliency corresponds to regions that contribute most to the overall spatial information score, typically by residing in a region of consistently low or high expression. Saliency here is defined as the accuracy with which a cell can be classified in repeated shuffled versus non-shuffled binary classification tasks.

then subtracting the two. A less trivial extension is to use Maxspin as an objective function in gating, clustering, or dimensionality reduction tasks. This adds a level of complexity because we would be simultaneously optimizing a function to assign cells to clusters (or to low-dimensional representations) and the discriminative bound on spatial information, but this is not so different from the setup in generative adversarial networks,[39] which have seen tremendous success despite there being a level of trickiness training such models.

By framing spatial coherence as spatial information and developing the tools for practical inference, we have presented a promising new perspective on spatial expression analysis and have taken the first step in exploring this idea by demonstrating a simple discriminative model that is more efficient and more accurate than state-of-the-art Gaussian process models at identifying SVGs across many settings. Though not a replacement in all cases for generative probabilistic models, it is, in many cases, a more accurate and efficient alternative that is designed to scale with spatial transcriptomics technology as it inevitably expands to very large datasets in the coming years.

Limitations of the study

Ultimately, "spatial coherence" or "spatially varying" expression can be subject to definitional disagreement. There may be different defensible answers to what constitutes a meaningful pattern of spatial organization. Other fields have grappled with these issues long before the recent relevance of spatial statistics in genomics. For example, the question of how best to measure segregation has been debated for decades in spatial demography.[40] The benchmarks presented here all rely on calling differential expression between identified transcriptionally distinct regions. While unlikely to capture all meaningful spatial variation, it is an intuitive baseline that we expect captures the most significant variations that should be uncontroversial as examples of SVGs. Further, these results hold up in our simulations in which the ground truth is known. Yet, for any given method, it is possible to design a benchmark that violates its assumptions and results in poor performance.

Maxpsin is designed with the intention of being run on a GPU, which adds some potential complication to using the software when compared with, for example, computing Moran's I. In a setting in which a GPU is unavailable and a dataset is very large, using Maxspin may be impractical. We hope the extensive benchmarks here will provide some guidance on a suitable alternative. If efficiency is critical, our results show that one can do much worse than Moran's I.

STAR★METHODS

Detailed methods are provided in the online version of this paper and include the following:

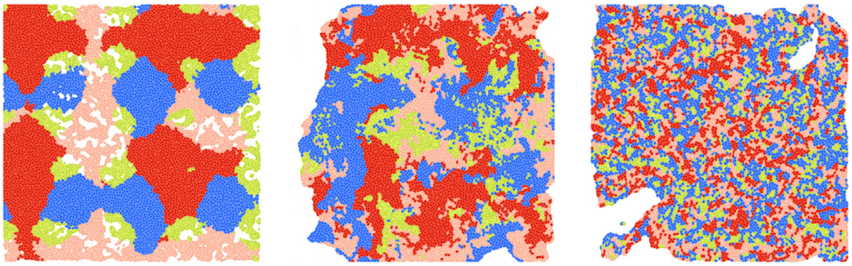

Figure 7. Cell positions and morphology are simulated using a cellular Potts model
Cells are initially arranged by type (here indicated by color) into highly ordered "blobs." Letting the cells migrate for an increasing number of iterations produces arrangements that are less globally ordered but still governed by a pairwise cell type adhesion matrix.

- KEY RESOURCES TABLE
- RESOURCE AVAILABILITY
  - Lead contact
  - Materials availability
  - Data and code availability
- METHOD DETAILS
  - Spatial auto-information
  - Approximating Jensen-Shannon divergence
  - Testing for statistical significance
  - Choosing neighborhood joint distribution
  - Choosing a classifier
  - Accounting for uncertainty in count data
  - Improving performance in sparse data by binning
  - Interpreting the spatial information score
  - Hierarchical clustering with pairwise spatial information
  - Saliency maps
  - Simulation of cell positions and morphology
  - Implementation
- QUANTIFICATION AND STATISTICAL ANALYSIS
  - Constructing ground truth spatially varying gene sets
  - Evaluating method performance

SUPPLEMENTAL INFORMATION

Supplemental information can be found online at https://doi.org/10.1016/j.crmeth.2023.100507.

ACKNOWLEDGMENTS

We are grateful to Edward Zhao for helpful feedback on the manuscript. This research was supported by funding from the National Institutes of Health (P01-CA225517 and U19AI128914, D.C.J., R.G., and E.W.N.), the Immunotherapy and Translational Data Science Integrated Research Centers at Fred Hutchinson Cancer Center to D.C.J., R.G., and E.W.N., the Fred Hutchinson Cancer Center New Development funds (E.W.N.), and The Andy Hill Endowment Distinguished Researcher CARE fund (E.W.N.). R.G.'s research was supported by institutional funding from the Faculté de Biologie et Medicine of the University of Lausanne and the Lausanne University Hospital.

AUTHOR CONTRIBUTIONS

D.C.J. implemented the method, performed the analysis, and wrote the manuscript. P.D., Y.K, and J.M.B. performed the CosMx spatial transcriptomics experiments. R.G. and E.W.N. supervised the project and wrote the manuscript.

DECLARATION OF INTERESTS

R.G. has received consulting income from Takeda and declares ownership in Ozette Technologies and Modulus Therapeutics. E.W.N. is a cofounder, advisor, and shareholder of ImmunoScape and is an advisor for Neogene Therapeutics and NanoString Technologies.

INCLUSION AND DIVERSITY

We support inclusive, diverse, and equitable conduct of research.

Received: February 6, 2023
Revised: April 3, 2023
Accepted: May 25, 2023
Published: June 16, 2023

REFERENCES

1. Marx, V. (2021). Method of the year: spatially resolved transcriptomics. Nat. Methods *18*, 9–14.
2. Moses, L., and Pachter, L. (2022). Museum of spatial transcriptomics. Nat. Methods *19*, 628.
3. Lundberg, E., and Borner, G.H.H. (2019). Spatial proteomics: a powerful discovery tool for cell biology. Nat. Rev. Mol. Cell Biol. *20*, 285–302.
4. Zhao, T., Chiang, Z.D., Morriss, J.W., LaFave, L.M., Murray, E.M., Del Priore, I., Meli, K., Lareau, C.A., Nadaf, N.M., Li, J., et al. (2022). Spatial genomics enables multi-modal study of clonal heterogeneity in tissues. Nature *601*, 85–91.
5. Ripley, B.D. (2005). Spatial Statistics (John Wiley & Sons).
6. Palla, G., Spitzer, H., Klein, M., Fischer, D., Schaar, A.C., Kuemmerle, L.B., Rybakov, S., Ibarra, I.L., Holmberg, O., Virshup, I., et al. (2022). Squidpy: a scalable framework for spatial omics analysis. Nat. Methods *19*, 171–178.
7. Miller, B.F., Bambah-Mukku, D., Dulac, C., Zhuang, X., and Fan, J. (2021). Characterizing spatial gene expression heterogeneity in spatially resolved single-cell transcriptomic data with nonuniform cellular densities. Genome Res. *31*, 1843–1855.
8. Svensson, V., Teichmann, S.A., and Stegle, O. (2018). SpatialDE: identification of spatially variable genes. Nat. Methods *15*, 343–346.
9. Sun, S., Zhu, J., and Zhou, X. (2020). Statistical analysis of spatial expression patterns for spatially resolved transcriptomic studies. Nat. Methods *17*, 193–200.
10. BinTayyash, N., Georgaka, S., John, S.T., Ahmed, S., Boukouvalas, A., Hensman, J., and Rattray, M. (2021). Non-parametric modelling of temporal and spatial counts data from RNA-seq experiments. Bioinformatics *37*, 3788–3795.
11. Weber, L.M., Saha, A., Datta, A., Hansen, K.D., and Hicks, S.C. (2022). nnSVG: scalable identification of spatially variable genes using nearest-neighbor Gaussian processes. Preprint at bioRxiv. https://doi.org/10.1101/2022.05.16.492124.

CellPress
OPEN ACCESS

12. Zhu, J., Sun, S., and Zhou, X. (2021). SPARK-X: non-parametric modeling enables scalable and robust detection of spatial expression patterns for large spatial transcriptomic studies. Genome Biol. *22*, 184.

13. Edsgärd, D., Johnsson, P., and Sandberg, R. (2018). Identification of spatial expression trends in single-cell gene expression data. Nat. Methods *15*, 339–342.

14. Anderson, A., and Lundeberg, J. (2021). sepal: identifying transcript profiles with spatial patterns by diffusion-based modeling. Bioinformatics.

15. Chang, Y., Liu, J., Ma, A., Li, Z., Liu, B., and Ma, Q. (2022). SpaGFT is a graph fourier transform for tissue module identification from spatially resolved transcriptomics. Preprint at bioRxiv. https://doi.org/10.1101/2022.12.10.519929.

16. Zhang, K., Feng, W., and Wang, P. (2022b). Identification of spatially variable genes with graph cuts. Nat. Commun. *13*, 5488.

17. Hu, J., Li, X., Coleman, K., Schroeder, A., Ma, N., Irwin, D.J., Lee, E.B., Shinohara, R.T., and Li, M. (2021). SpaGCN: integrating gene expression, spatial location and histology to identify spatial domains and spatially variable genes by graph convolutional network. Nat. Methods *18*, 1342–1351.

18. Zhang, C., Dong, K., Aihara, K., Chen, L., and Zhang, S. (2022a). STAMarker: determining spatial domain-specific variable genes with saliency maps in deep learning. Preprint at bioRxiv. https://doi.org/10.1101/2022.11.07.515535.

19. Dries, R., Zhu, Q., Dong, R., Eng, C.-H.L., Li, H., Liu, K., Fu, Y., Zhao, T., Sarkar, A., Bao, F., et al. (2021). Giotto: a toolbox for integrative analysis and visualization of spatial expression data. Genome Biol. *22*, 78.

20. Liu, Q., Hsu, C.-Y., and Shyr, Y. (2022). Scalable and model-free detection of spatial patterns and colocalization. Genome Res. *32*, 1736–1745.

21. Tschannen, M., Djolonga, J., Rubenstein, P.K., Gelly, S., and Lucic, M. (2019). On mutual information maximization for representation learning. Preprint at arXiv. https://doi.org/10.48550/arXiv.1907.13625.

22. Belghazi, M.I., Baratin, A., Rajeswar, S., Ozair, S., Bengio, Y., Courville, A., and Devon Hjelm, R. (2018). MINE: mutual information neural estimation. Preprint at arXiv. https://doi.org/10.48550/arXiv.1801.04062.

23. Graner, F., and Glazier, J.A. (1992). Simulation of biological cell sorting using a two-dimensional extended potts model. Phys. Rev. Lett. *69*, 2013–2016.

24. Niculescu, I., Textor, J., and de Boer, R.J. (2015). Crawling and gliding: a computational model for Shape-Driven cell migration. PLoS Comput. Biol. *11*, e1004280.

25. Maynard, K.R., Collado-Torres, L., Weber, L.M., Uytingco, C., Barry, B.K., Williams, S.R., Catallini, J.L., 2nd, Tran, M.N., Besich, Z., Tippani, M., et al. (2021). Transcriptome-scale spatial gene expression in the human dorsolateral prefrontal cortex. Nat. Neurosci. *24*, 425–436.

26. Vizgen. (2022). MERFISH FFPE human immuno-oncology data set. https://info.vizgen.com/merscope-ffpe-solution.

27. He, S., Bhatt, R., Brown, C., Brown, E.A., Buhr, D.L., Chantranuvatana, K., Danaher, P., Dunaway, D., Garrison, R.G., Geiss, G., et al. (2022). High-plex multiomic analysis in FFPE at subcellular level by spatial molecular imaging. Preprint at bioRxiv. https://doi.org/10.1101/2021.11.03.467020.

28. Breda, J., Zavolan, M., and van Nimwegen, E. (2019). Bayesian inference of the gene expression states of single cells from scRNA-seq data. Preprint at bioRxiv. https://doi.org/10.1101/2019.12.28.889956.

29. Traag, V.A., Waltman, L., and van Eck, N.J. (2019). From louvain to leiden: guaranteeing well-connected communities. Sci. Rep. *9*, 5233.

30. Stewart, B.J., Ferdinand, J.R., Young, M.D., Mitchell, T.J., Loudon, K.W., Riding, A.M., Richoz, N., Frazer, G.L., Staniforth, J.U.L., Vieira Braga, F.A., et al. (2019). Spatiotemporal immune zonation of the human kidney. Science *365*, 1461–1466.

31. Chuang, S.-T., Patton, K.T., Schafernak, K.T., Papavero, V., Lin, F., Baxter, R.C., Teh, B.T., and Yang, X.J. (2008). Over expression of insulin-like growth factor binding protein 3 in clear cell renal cell carcinoma. J. Urol. *179*, 445–449.

32. Miess, H., Dankworth, B., Gouw, A.M., Rosenfeldt, M., Schmitz, W., Jiang, M., Saunders, B., Howell, M., Downward, J., Felsher, D.W., et al. (2018). The glutathione redox system is essential to prevent ferroptosis caused by impaired lipid metabolism in clear cell renal cell carcinoma. Oncogene *37*, 5435–5450.

33. Williams, A.A., Higgins, J.P.T., Zhao, H., Ljunberg, B., and Brooks, J.D. (2009). CD 9 and vimentin distinguish clear cell from chromophobe renal cell carcinoma. BMC Clin. Pathol. *9*, 9.

34. Kutty, R.G., Talipov, M.R., Bongard, R.D., Lipinski, R.A.J., Sweeney, N.L., Sem, D.S., Rathore, R., and Ramchandran, R. (2017). Dual specificity phosphatase 5-substrate interaction: a mechanistic perspective. Compr. Physiol. *7*, 1449–1461.

35. Yokoyama, Y., Grünebach, F., Schmidt, S.M., Heine, A., Häntschel, M., Stevanovic, S., Rammensee, H.-G., and Brossart, P. (2008). Matrilysin (MMP-7) is a novel broadly expressed tumor antigen recognized by antigen-specific T cells. Clin. Cancer Res. *14*, 5503–5511.

36. Nirschl, T.R., El Asmar, M., Ludwig, W.W., Ganguly, S., Gorin, M.A., Johnson, M.H., Pierorazio, P.M., Drake, C.G., Allaf, M.E., and Zarif, J.C. (2020). Transcriptional profiling of tumor associated macrophages in human renal cell carcinoma reveals significant heterogeneity and opportunity for immunomodulation. Am. J. Clin. Exp. Urol. *8*, 48–58.

37. Peranzoni, E., Lemoine, J., Vimeux, L., Feuillet, V., Barrin, S., Kantari-Mimoun, C., Bercovici, N., Guérin, M., Biton, J., Ouakrim, H., et al. (2018). Macrophages impede CD8 T cells from reaching tumor cells and limit the efficacy of anti–PD-1 treatment. Proc. Natl. Acad. Sci. USA *115*, E4041–E4050.

38. Reimold, A.M., Iwakoshi, N.N., Manis, J., Vallabhajosyula, P., Szomolanyi-Tsuda, E., Gravallese, E.M., Friend, D., Grusby, M.J., Alt, F., and Glimcher, L.H. (2001). Plasma cell differentiation requires the transcription factor XBP-1. Nature *412*, 300–307.

39. Goodfellow, I., Pouget-Abadie, J., Mirza, M., Xu, B., Warde-Farley, D., Ozair, S., Courville, A., and Bengio, Y. (2014). Generative adversarial nets. Preprint at arXiv. https://doi.org/10.48550/arXiv.1406.2661.

40. Wong, D.W. (2016). From aspatial to spatial, from global to local and individual: are we on the right track to spatialize segregation measures? In Recapturing Space: New Middle-Range Theory in Spatial Demography, F.M. Howell, J.R. Porter, and S.A. Matthews, eds. (Springer International Publishing), pp. 77–98.

41. Hao, Y., Hao, S., Andersen-Nissen, E., Mauck, W.M., 3rd, Zheng, S., Butler, A., Lee, M.J., Wilk, A.J., Darby, C., Zager, M., et al. (2021). Integrated analysis of multimodal single-cell data. Cell *184*, 3573–3587.e29. https://doi.org/10.1016/j.cell.2021.04.048.

42. Hjelm, R.D., Fedorov, A., Lavoie-Marchildon, S., Grewal, K., Bachman, P., Trischler, A., and Bengio, Y. (2018). Learning deep representations by mutual information estimation and maximization. Preprint at arXiv. https://doi.org/10.48550/arXiv.1808.06670.

43. Altieri, L., Cocchi, D., and Roli, G. (2018). A new approach to spatial entropy measures. Environ. Ecol. Stat. *25*, 95–110.

44. Kipf, T.N., and Welling, M. (2016). Semi-Supervised classification with graph convolutional networks. Preprint at arXiv. https://doi.org/10.48550/arXiv.1609.02907.

45. Tsai, Y.-H.H., Ma, M.Q., Yang, M., Zhao, H., Morency, L.-P., and Salakhutdinov, R. (2021). Self-supervised representation learning with relative predictive coding. Preprint at arXiv. https://doi.org/10.48550/arXiv.2103.11275.

46. Nowozin, S., Cseke, B., and Tomioka, R. (2016). F-GAN: Training generative neural samplers using variational divergence minimization. Adv. Neural Inf. Process. Syst. *29*, 271–279.

47. Brakel, P., and Bengio, Y. (2017). Learning independent features with adversarial nets for non-linear ICA. Preprint at arXiv. https://doi.org/10.48550/arXiv.1710.05050.

48. Kingma, D.P., and Ba, J. (2014). Adam: a method for stochastic optimization. Preprint at arXiv. https://doi.org/10.48550/arXiv.1412.6980.

49. Smola, A.J., and Kondor, R. (2003). Kernels and regularization on graphs. In Learning Theory and Kernel Machines (Springer Berlin Heidelberg), pp. 144–158.

50. Simonyan, K., Vedaldi, A., and Zisserman, A. (2013). Deep inside convolutional networks: visualising image classification models and saliency maps. Preprint at arXiv. https://doi.org/10.48550/arXiv.1312.6034.

51. Bradbury, J., Frostig, R., Hawkins, P., Johnson, M.J., Leary, C., Maclaurin, D., Necula, G., Paszke, A., VanderPlas, J., Wanderman-Milne, S., and Zhang, Q. (2018). JAX: composable transformations of Python+NumPy programs. http://github.com/google/jax.

52. Heek, J., Levskaya, A., Oliver, A., Ritter, M., Rondepierre, B., Steiner, A., and van Zee, M. (2020). Flax: a neural network library and ecosystem for JAX. http://github.com/google/flax.

STAR★METHODS

KEY RESOURCES TABLE

REAGENT or RESOURCE	SOURCE	IDENTIFIER
Deposited data		
CosMx Renal Cell Carcinoma dataset	This paper	https://doi.org/10.6084/m9.figshare.21539253.v1
Software and algorithms		
maxspin version 0.1.1	This paper	https://doi.org/10.5281/zenodo.7901114
Cronenberg.jl version 0.0.1	This paper	https://doi.org/10.5281/zenodo.7901437
Seurat version 4.3.0	Hao, et al.[41]	https://github.com/satijalab/seurat
SpaGene version 0.1.0	Liu, et al.[20]	https://github.com/liuqivandy/SpaGene
SPARK version 1.1.1	Sun, et al.[30], Zhu, et al.[4]	https://github.com/xzhoulab/SPARK
Giotto version 1.1.1	Dries, et al.[19]	https://github.com/drieslab/Giotto
SpatialDE version 2.0.0	Svensson, et al.[9]	https://github.com/Teichlab/SpatialDE
nnSVG version 1.2.0	Weber, et al.[26]	https://github.com/lmweber/nnSVG
scGCO version 1.1.2	Zhang, et al.[18]	https://github.com/WangPeng-Lab/scGCO
SpaGFT version 0.1.1	Chang, et al.[15]	https://github.com/jxLiu-bio/SpaGFT
GPcounts version 0.1	BinTayyash, et al.[10]	https://github.com/ManchesterBioinference/GPcounts
trendsceek version 1.0.0	Edsgärd, et al.[13]	https://github.com/edsgard/trendsceek

RESOURCE AVAILABILITY

Lead contact

Further information and requests for resources and reagents should be directed to and will be fulfilled by the lead contact, Daniel Jones (djones3@fredhutch.org)

Materials availability

This study did not generate new unique reagents.

Data and code availability

- CosMx SMI data have been deposited at Figshare and are publicly available as of the date of publication. DOIs are listed in the key resources table.
- All original code has been deposited at GitHub and is publicly available as of the date of publication. DOIs are listed in the key resources table.
- Any additional information required to reanalyze the data reported in this paper is available from the lead contact upon request.

METHOD DETAILS

Spatial auto-information

The concept of spatial auto-correlation has been studied for decades using metrics like Moran's I.[5] Our goal is to create a more general and flexible notion spatial dependence by instead adopting an information theoretic perspective and considering auto-mutual information.

We score the "spatial coherence" of a gene's expression by approximating the spatial auto-mutual information over nearby expression values. Let $p(x)$ be the marginal expression distribution for the gene of interest, $p_N(x,x')$ the joint distribution over pairs of expression values occurring in the same spatial neighborhood. The *spatial auto-mutual information* is then

$$I_N(x,x') := D_{\mathrm{KL}}(p_N(x,x') \| p(x)p(x')) \quad \text{(Equation 1)}$$

$$= \int_x \int_{x'} p_N(x,x') \log \frac{p_N(x,x')}{p(x)p(x')} dx' dx \quad \text{(Equation 2)}$$

where D_{KL} is the Kullback-Leibler divergence.

Similar to,[42] we found that using Jensen-Shannon divergence in place of Kullback-Leibler divergence lead to somewhat better results. The resulting objective function can be thought of as a non-standard version of mutual information, which we refer to here simply as *spatial information*. It can also be rewritten as standard mutual information, though no longer between neighboring expression values, but instead the mutual information between observed pairs of expression values and 0–1 binary variable z indicating whether they have been shuffled or not. More formally, if we let $(\tilde{x}, \tilde{x}')$ be drawn from a mixture distribution $zp_N(x,x') + (1 - z)p(x)p(x')$, then the objective can be written as

$$D_{\mathrm{JS}}(p_N(x,x') \| p(x)p(x')) = I_N((\tilde{x},\tilde{x}') \| z) \quad \text{(Equation 3)}$$

Both of these definitions hinge on how "spatial neighborhood" is defined. In the simplest form, we can consider two cells/spots neighbors if they are within some distance ϵ. If $s, s' \in \mathbb{R}^d$ are spatial coordinates corresponding to x, x', respectively, we can marginalize out the spatial coordinates within the same neighborhood to arrive at a joint neighborhood expression distribution on fixed radius neighborhoods

$$p_N(x,x') = \int_s \int_{s' \in B_\epsilon(s)} p(x,s)p(x',s')ds'ds \quad \text{(Equation 4)}$$

This can be generalized by defining soft neighborhoods using a joint distribution over pairs of spatial positions

$$p_N(x,x') = \int_s \int_{s'} p(x|s)p(x'|s')p_N(s,s')ds'ds \quad \text{(Equation 5)}$$

where $p_N(s,s')$ is chosen to assign higher probability to nearby coordinates. To recover Equation 4, we can make it a uniform distribution over pairs within distance ε of each other.

A related notion of spatial mutual information occurs in Altieri, et al.[43] There, spatial mutual information is defined simply as the mutual information between a pair of observed values (x, x') and their spatial distance $d(s, s')$, both of which are discretized in their setting. This is seemingly more straightforward as it avoids having to define a neighborhood distribution, but in a large dataset training on all n^2 pairs would be inefficient, and randomly sampled subsets of pairs will tend to be mostly distant. So in practice, this is potentially harder to estimate. Furthermore, the precise distance between a pair of cell is unlikely to be meaningful when those cells are far away from each other.

Another possible definition is to consider the mutual information between a single expression value and its entire spatial neighborhood. This may enable the detection of more elaborate spatial patterns, but has practical issues. Neighborhoods could easily be memorized by a model, overestimating mutual information, so the joint distribution would have to be very carefully modeled to avoid overfitting. We would also have to aggregate information across every node's neighborhood, using for example a graph neural network,[44] at every step of training and evaluation. The computational cost would thus limit how large of a neighborhood we could consider.

Computing either the JS or KL divergence has two major hurdles in practical applications. First, it necessitates finding a reasonable model for the joint and marginal probabilities. An overparameterized model for $p_N(x,x')$ risks memorizing the data or otherwise discovering spatial patterns that are too subtle to be meaningful, whereas an insufficiently expressive model will fail to find true patterns. Second, even with a suitable model in hand, we still have to compute the integral in Equation 2. In this work, we sidestep both of these issues by instead estimating a lower bound on the JS divergence using a simple discriminative model.

Approximating Jensen-Shannon divergence

A number of recent studies have focused on avoiding the problem of computing mutual information directly by deriving more tractable lower bounds.[21] These formalize the intuition that the easier it is to distinguish random draws form the joint distribution from random draws from the marginals, the higher the mutual information must be. Or, in the context of spatial transcriptomics: a gene's expression is spatially coherent if we can, with some reliability, predict whether a pair of expression values was drawn at uniform, or drawn from nearby cells.

A number of bounds on different divergence measures have been derived (for a review and comparison, see Tsai, et al.[45]). For this work, we found the bound on Jensen-Shannon divergence proposed by Nowozin, et al.[46] to work well, which is equivalent to the binary cross-entropy objective used by Brakel, et al.[47] A Jensen-Shannon based variant of the standard mutual information can be defined as the Jensen-Shannon divergence between $p_N(x,x')$ and $p(x)p(x')$, and bounded below by

$$\begin{aligned} I_{\mathrm{JS}}(x,x') &= D_{\mathrm{JS}}(p_N(x,x') \| p(x)p(x')) \\ &\geq \max_\theta \mathbb{E}_{p_N(\cdot,\cdot)}[-\sigma(-f_\theta(x,x'))] - \mathbb{E}_{p(\cdot)p(\cdot)}[\sigma(f_\theta(x,x'))] \end{aligned} \quad \text{(Equation 6)}$$

where $\sigma(x) := \log(1 + \exp(x))$ is the softplus function, and f_θ is a classifier function with parameters θ. Both expectations are taken over (x, x') with respect to either the joint neighborhood distribution or the product of the marginals.

Intuitively, we want to train f_θ to distinguish samples drawn from the same neighborhood, using p_N, and pairs that are independent draws from across the sample. The better it is able to distinguish these, the higher the lower bound on the Jensen-Shannon mutual information. Thus, expression patterns are spatially coherent to the degree that we can learn a pattern in nearby pairs.

Assuming we can efficiently sample from p and p_N, and f_θ is differentiable with respect to θ, this objective can be easily optimized using stochastic gradient descent, by drawing samples from the joint and marginals at each iteration, computing the objective in Equation 6, and backpropagating gradients to update θ.

Concretely, each step of the optimization algorithm proceeds as follows, where $x = (x_1, \ldots, x_n)$ is a single gene's expression measured across n cells or spots, and $s = (s_1, \ldots, s_n)$ is their spatial positions.

$I \leftarrow (\text{randomwalk}_k(i)\ \text{for}\ i = 1, \ldots, n)$ ▷ k-step random walk from each cell
$x' \leftarrow (x_{I_1}, \ldots, x_{I_n})$ ▷ Expression at neighboring cells
$\tilde{x} \leftarrow \text{shuffle}(x)$ ▷ Randomly shuffle values across cells
$\tilde{x}' \leftarrow (\tilde{x}_{I_1}, \ldots, \tilde{x}_{I_n})$. ▷ Neighboring expression in shuffled data
$d \leftarrow (\|s_i - s_{I_i}\|_2\ \text{for}\ i = 1, \ldots, n)$ ▷ Euclidean distance of each random walk

$$\theta \leftarrow \theta + \gamma \nabla_\theta \left(-\sum_{i=1}^{n} \sigma\left(-f_\theta\left(x_i, x_i', d_i\right)\right) - \sum_{i=1}^{n} \sigma\left(f_\theta\left(\tilde{x}_i, \tilde{x}_i', d_i\right)\right) \right)$$

Here γ is the step size. In practice we use the Adam[48] optimizer, rather than a fixed step size.

Testing for statistical significance

Because Maxspin is based on attempting a binary classification between shuffled and non-shuffled, it is trivial to construct a null-hypothesis statistical test in addition to quantifying spatial information. The natural null hypothesis is that the classifier can do no better than random guessing, and thus the number of correctly classified examples k is drawn from a $\text{Binomial}(n, 0.5)$ distribution. The p value is then computed from the CDF of the Binomial distribution.

Most genes will either have low expression or display some level of spatial organization, if the spatial context examined large enough. Because of this, examining spatial information will often be more informative than binarizing into SVG or non-SVG using a null hypothesis test. This is doubly true for reasons of numerical precision: p values computed for highly spatially organized genes will tend to saturate floating-point arithmetic and produce many zeros. As noted, SpatialDE, among other methods, suffered from this phenomenon, reducing its performance in our benchmarks.

Choosing neighborhood joint distribution

The definition of "spatial neighborhood" is determined by the neighborhood joint distribution p_N. Optimizing the Jensen-Shannon lower bound using stochastic gradient descent necessitates a distribution that can be efficiently sampled from. In Equation 5 we showed how joint neighborhood expression can be defined in terms of a joint distribution over pairs of positions $p(s, s')$ defining a soft neighborhood, where s, s' are spatial positions of cells.

In Maxspin, we use the distribution induced by considering random walks of a fixed length between s and s'. A neighborhood graph can be formed by Delaunay triangulation, fixed distance neighborhoods, or according to a grid in the case of Visium data. From this graph, a transition matrix is formed where each neighbor is visited with equal probability. We can then define the joint distribution over positions as

$$p(s, s') := p_k(s'|s)p(s)$$

where $p(s)$ is a uniform distribution over cell positions, and $p_k(s'|s)$ is the probability of starting at the cell at position s and arriving at the cell at position s' after k random steps.

Larger values of k will consider a larger neighborhood, and thus be more tuned to finding larger scale spatial patterns, and a small k will be more tuned to finding small scale patterns. Throughout this paper we used $k = 10$, finding the results to be not particularly sensitive to this parameter, so long as it was not very small (less than 3). The limiting stationary distribution is a uniform distribution over pairs of nodes, so at a certain point performance will degrade if k is too large, but the computational burden of sampling such long walks becomes an issue before that point.

This random walk distribution is largely equivalent to the p-step random walk kernel discussed by Smola, et al.,[49] which is proposed as a more practical alternative to their graph diffusion kernel.

It has yet to be established definitively whether definitions of spatial locality that are graph based, as used here, or distance based, as used for most Gaussian process covariance functions, are a better fit for spatial transcriptomics in general. Graph based approaches are simpler though, since there is no need to try to calibrate the scale of the kernel function to the spatial coordinates which come in a variety of units (pixels, grid indices, micrometers, etc). As with common distance based kernel functions, random walk based kernels also emphasize nearness, as a walk between two distant cells is less probable.

Choosing a classifier

Bounding JS divergence using a classifier solves the issue of having to model the joint probability distribution. Classifier models, typically conceptualized as a distribution over labels conditioned on observations, are typically far easier to model than full generative probabilistic models. Framing the problem as classification also presents an opportunity to remedy another major weakness of information theoretic methods: since divergence captures any non-independence relationship it can find relationships that are spurious or uninteresting. In principle, any differentiable classifier can be used for f_θ, but too powerful a learner, like a deep neural network, risks memorizing the data, or identifying patterns too subtle or elaborate to be of interest. In practice we need a learner than is sufficiently weak, yet able to capture the kinds of patterns that are of potential scientific interest.

Since we are most often interested in the phenomenon of a gene being expressed similarly in spatial neighborhood, we use a simple classifier of the form

$$f_{w_s,w_d,b,s}(x,x') := w_s|x+x'+s| - w_d|x-x'|+b \quad \text{(Equation 7)}$$

We constrain the weights $w_s, w_d \in \mathbb{R}^+$ to be positive using a softplus transformation. The intuition here is that we assign high scores to node pairs that are similarly expressed (captured by the $w_d|x-x'|$ term) and both either unusually low or high (captured by the $w_s|x+x'+s|$ term). Dropping the positivity constraint would allow the detection of anti-correlation, which could potentially be of interest, but more often we are interested in positive correlation, and restricting the signs of the weight slightly improves performance for such cases.

We found this function family to be suitably general to discover simple patterns of low or high expression regions, but in other settings where the goal is to find more elaborate spatial patterns, the method can be trivially adapted by choosing a more powerful classifier. The risk of doing so is to overfit or memorize the data, but this can be further remedied with standard regularization techniques.

We further allow the classifier weight these scores according to the distance of the random walk, by passing the distance of the random walk to classifier for both the neighborhood and uniform sampled pair. This allows it to discount long walks when the spatial organization is very local. To do this we weight classifier scores by $\exp(-d/\alpha)$, where d is the Euclidean distance of the walk, and α is a parameter that is learned on a per-gene basis. The weighted classifier function then takes the form

$$f_{w_s,w_d,b,s,\alpha}(x,x',d) := \exp(-d/\alpha) f_{w_s,w_d,b,s}(x,x') \quad \text{(Equation 8)}$$

Optimization is over the set of parameters $\theta = (w_s, w_d, b, s, \alpha)$, each a real number.

Accounting for uncertainty in count data

Discrete and often sparse count data represents a noisy estimate of gene expression. Methods often fail to account for this source of uncertainty resulting overconfident predictions. Instead of training on raw, or transformed count data, we instead resample expression values periodically during stochastic gradient descent, effectively optimizing an expectation over unobserved expression values. This has the concrete effect of minimizing the possibility of overfitting.

With count data, we can either assume counts represent an unconfounded measurement of absolute expression, or we can assume that the total counts per cell/spot represent a confounding variable and instead deal in proportional expression (e.g. "transcripts per million"). In the former case, we adopt a Gamma-Poisson model, and train on values sampled from the posterior Gamma distribution at each iteration. When treating data proportionally, we adopt a Dirichlet-Multinomial model and train on samples drawn from the posterior Dirichlet distribution.

The same approach can be used with a more sophisticated probabilistic model, by running Maxspin on samples drawn from its posterior. We took this approach in our analysis of the CosMx RCC data. Posterior inference done by Sanity[28] provides posterior means and standard deviations. We used these values to sample expression values from a normal distribution when running Maxspin. In the future, other types of models can be used to handle isoform- or allele-specific expression by accounting for uncertainty over read assignment.

Improving performance in sparse data by binning

When expression for a gene is very sparse, training can be inefficient, because it relies on the relatively small proportion of random walks between cells with nonzero expression. To remedy this, we bin data at multiple resolutions, and train jointly on the original data and the binned data.

Binning is nontrivial because if bins end up with differing numbers of cells, it can introduce subtle statistical dependencies which can falsely manifest as positive spatial information scores, even if normalizing for cell count. To overcome this, we spatially bin data using a modified kd-tree. For n cells/spots and a specified bin size k, we first discard a random $n \bmod k$ cells to ensure the data can be exactly binned. We then recursively split along alternating axes, ensuring at each split that both partitions can be divided exactly by k, until we reach partitions of size k. The result is spatially arranged bins each with exactly k cells. By default we include binned data with bin sizes $k = 4, 8, 16$ when training. Of course, the result of binning is fewer bins than there were cells, so when training upweight the objective function for each binning by $\sqrt{k}$. Weighting offers a trade-off. More highly weighted large bins will make the method more sensitive to broad but sparse patterns, but somewhat less sensitive to very small scale patterns.

Interpreting the spatial information score

The spatial information score computed by Maxspin is a value $s_i \in [0,1]$ for each gene i quantifying the accuracy of predicting whether a pair of cells was sampled from a random walk in its neighborhood or uniformly at random. Though its interpretation may not be immediately obvious, it has some advantages over correlations or p values computed by other methods.

Most critically, unlike every other method considered, the score is computed on a per-cell basis. The score for a gene i is simply the sum of the scores for each cell j. That is, $s_i = \sum s_{ij}$. Because of this, it is trivial to determine why a gene was assigned a high score, whereas with every other existing method, some deeper analysis has to be performed. In some instances, taking the mean might be more appropriate, but by default we do not try to control for the number of cells. A spatially coherent pattern involving more cells will score higher than a similar pattern involving fewer.

These per-cell scores do have a simple interpretation as the log-probability assigned to the true labels in the classified pairs, normalized to a baseline of a random guessing. To see this we simply have to rewrite the objective function. First consider the objective function

$$s_{ij} = \mathbb{E}_{(x_n,x_n'),(x_f,x_f')}\left[-\sigma\left(-f_\theta(x_n,x_n')\right) - \sigma\left(f_\theta(x_f,x_f')\right)\right]$$

where σ is the softplus function, and the expectation is taken over the random walk distribution $(x_n,x_n') \sim p_N(\cdot,\cdot)$ as well as the uniform sampling distribution $(x_f,x_f') \sim p(\cdot)p(\cdot)$, with the mnemonic n for near and f for far. This can be rewritten in terms of the sigmoid function $\sigma(x) = 1/(1+\exp(-x))$.

$$\begin{aligned} s_{ij} &= \mathbb{E}_{(x_n,x_n'),(x_f,x_f')}\left[\log\left(\frac{1}{1+\exp\left(-f_\theta(x_n,x_n')\right)}\right) + \log\left(\frac{1}{1+\exp\left(f_\theta(x_f,x_f')\right)}\right)\right] \\ &= \mathbb{E}_{(x_n,x_n'),(x_f,x_f')}\left[\log\sigma\left(f_\theta(x_n,x_n')\right) + \log\sigma\left(-f_\theta(x_f,x_f')\right)\right] \\ &= \mathbb{E}_{(x_n,x_n'),(x_f,x_f')}\left[\log\left(\sigma\left(f_\theta(x_n,x_n')\right)\left(1-\sigma\left(f_\theta(x_f,x_f')\right)\right)\right)\right] \end{aligned}$$

If we interpret $\sigma(f_\theta(\cdot))$ as the probability assigned by the classifier, we can see that this spatial information score is simply the log probability of assigning true labels to both the near and far examples. Each cell's score is then a score in $(-\infty, 0.0]$.

Random guessing would result in a baseline score of $\log(0.5 \cdot 0.5) \approx -1.38$, so scores in practice are in $[\log(0.25), 0]$, only occasionally lying slightly below due to numerical imprecision. Maxspin further normalizes these by shifting and and scaling these to lie in $[0,1]$, allowing them to be loosely interpreted similarly to correlations, though typically much smaller, since the classifier rarely approaches perfect accuracy. Summing across cells we then have per-gene scores s_i than lie in $[0,n]$, and can be divided by n if so desired.

Hierarchical clustering with pairwise spatial information

Pairwise information scores were computed and hierarchical clustering was run with with complete linkage. Pairwise information, unlike distance larger for more closely related genes. To use with hierarchical clustering, which assumes pairwise distances, pairwise information I_{ij} is first symmetrized as $\tilde{I}_{ij} = (I_{ij} + I_{ji})/2)$ to reduce numerical inaccuracy, then transformed into a distance as

$$D_{ij} = \frac{1}{(1+\tilde{I}_{ij})^s}$$

where s is a constant controlling the relative scaling, which we set to $s = 0.5$.

Saliency maps

Our spatial information score, which captures the aggregate classifier performance, can be disaggregated to cell-level scores which can be used to determine which regions are spatial coherent. The resulting images are analogous to saliency maps proposed by Simonyan et al.[50] to visualize which regions of an image contribute disproportionately in convolutional neural network classifiers. Both here and in that work, saliency maps provide an explanation to go along with the prediction, which can be useful for diagnosis, or in the future, perhaps for clustering and other spatial analysis tasks.

Figure 6 shown examples of spatially varying genes detected in the CosMx RCC dataset and their accompanying saliency maps.

Simulation of cell positions and morphology

Our Cellular Potts model simulates cell position and morphology, with cell migrating for some number of iterations. More iterations will tend to produce less simplistic spatial patterns, white retaining a non-random configuration due to random pairwise cell type adherence. Figure 7 shows an example of the same simulation being run increasing numbers of iterations.

The simulations taken from SPARK-X are comparatively much simpler, with fixed, simple patterns of spatial variation (Figures S1C and S1D).

Implementation

The method in implemented in Python using jax[51] and flax,[52] and built on data structures from squidpy.[6]

QUANTIFICATION AND STATISTICAL ANALYSIS

Constructing ground truth spatially varying gene sets

Across the benchmarks we used the same strategy to construct a set of high confidence spatially varying genes to use as a proxy for ground truth. In each dataset we annotated distinct spatial regions and then did pairwise differential expression tests between regions using the Wilcoxon signed rank test. p-values were adjusted using the Benjamini-Hochberg procedure. We then selected genes with adjusted p values below 0.01. This analysis was performed in R, using Seurat.[41]

Evaluating method performance

Methods approach the problem of discovering spatially varying genes differently. Some methods produce p values, but others posterior probabilities or autocorrelation scores. Therefore we do not seek to evaluate how well calibrated p values or posterior probabilities are, and instead performed an analysis based on rank. Regardless of how the method approaches the problem, it ought to be able to rank genes so that the most those with the most spatially organized expression come out on top.

Statistical analysis for the benchmark comparisons between methods was based on area under the precision recall curve (PR-AUC). Since performance varied dramatically between benchmarks, we normalized these scores by subtracting the PR-AUC score of Moran's I, which we consider a reasonable default method for spatial autocorrelation, producing a summary score we call Δ PR-AUC, measuring to what degree a method under- or overperforms Moran's I.

Cell Reports Methods, Volume *3*

Supplemental information

An information theoretic approach to detecting spatially varying genes

Daniel C. Jones, Patrick Danaher, Youngmi Kim, Joseph M. Beechem, Raphael Gottardo, and Evan W. Newell

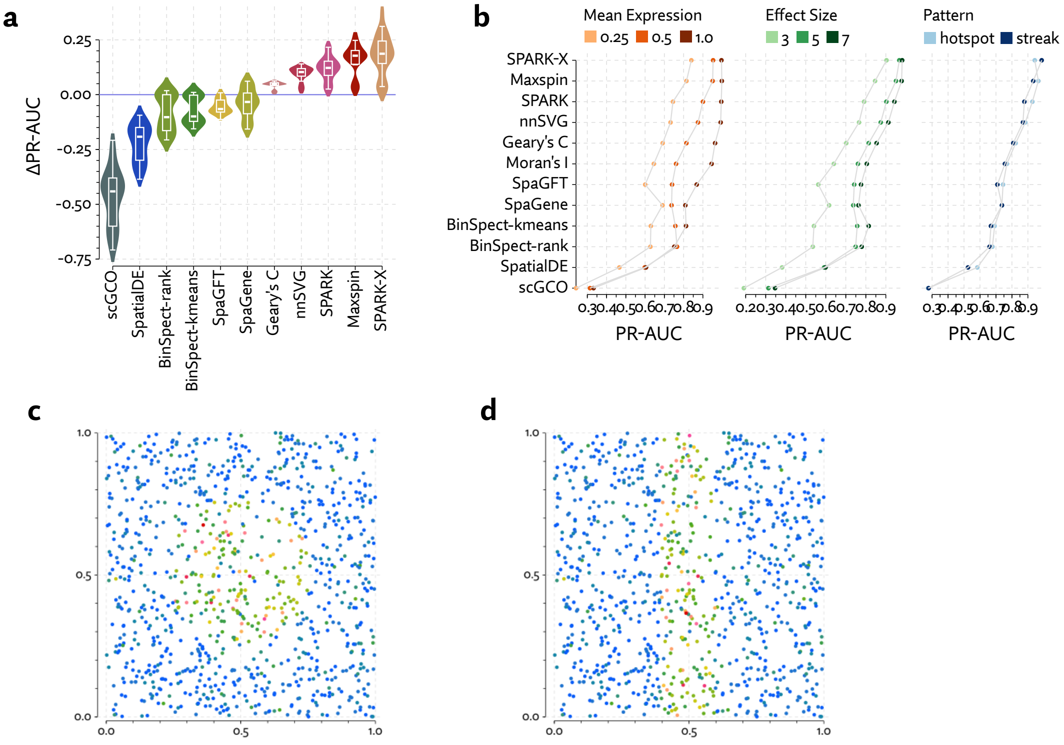

Figure S1: **An alternative simulation benchmark, related to Figure 2.** A simulation consisting uniformly distributed cells with a circle ("hotspot") or vertical band ("streak") of perturbed expression. **a** Aggregate performance is measured by computing the area under the precision-recall curve and subtracting the value obtained by Moran's I. **b** Performance is further broken down by simulation parameters.

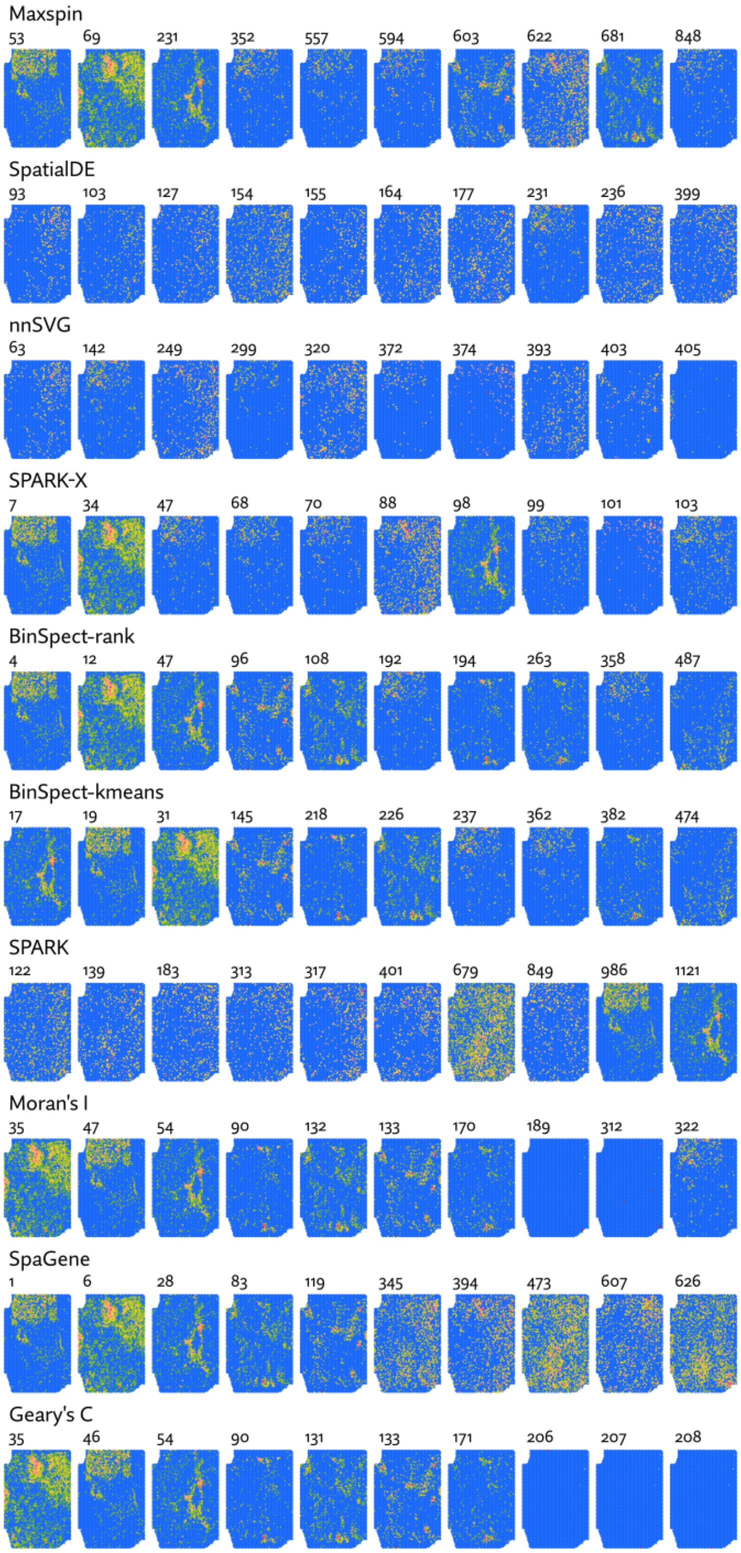

Figure S2: **A comparison of the top-10 highest ranking false-positives across tested methods, related to Figure 3**. Top ranking genes for each method were taken from sample 151509 from the Visium dataset. Listed above each example is it's rank. Many of the same examples appear across methods, but tend be ranked lower by Maxspin.

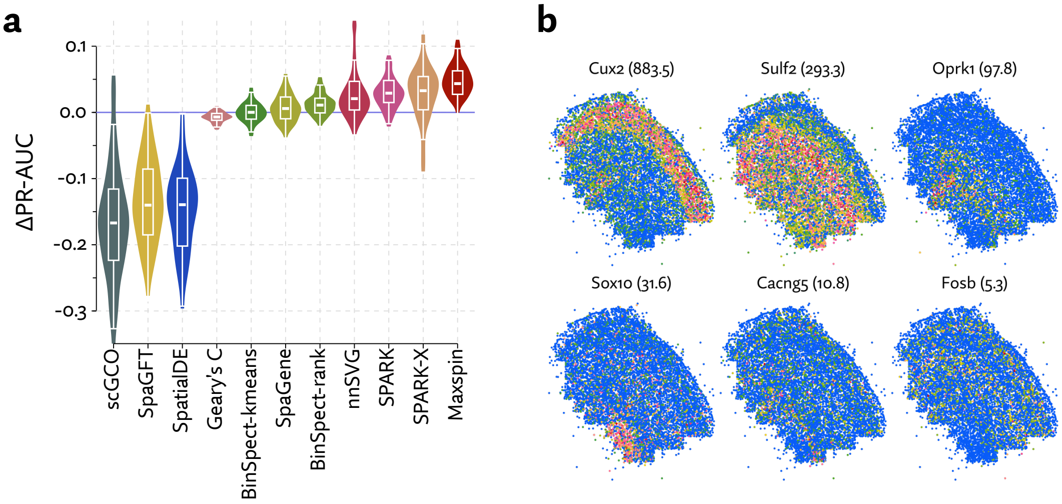

Figure S3: **Additional MERFISH benchmarking, related to Figure 3.** Mouse primary motor cortex MERFISH data was taken from Zhang, et al.[1]. The data consists of panel of 252 genes measured in 12 experiments, each consisting of a number of coronal slices (64 in total), with a total of 280,186 cells. We annotated cortical layers by reproducing the procedure described by the authors, discarding one of the experiments where we could not reliably annotate the L6b layer. (a) results are shown for each applicable method, and (b) examples of SVGs with their accompanying information scores.

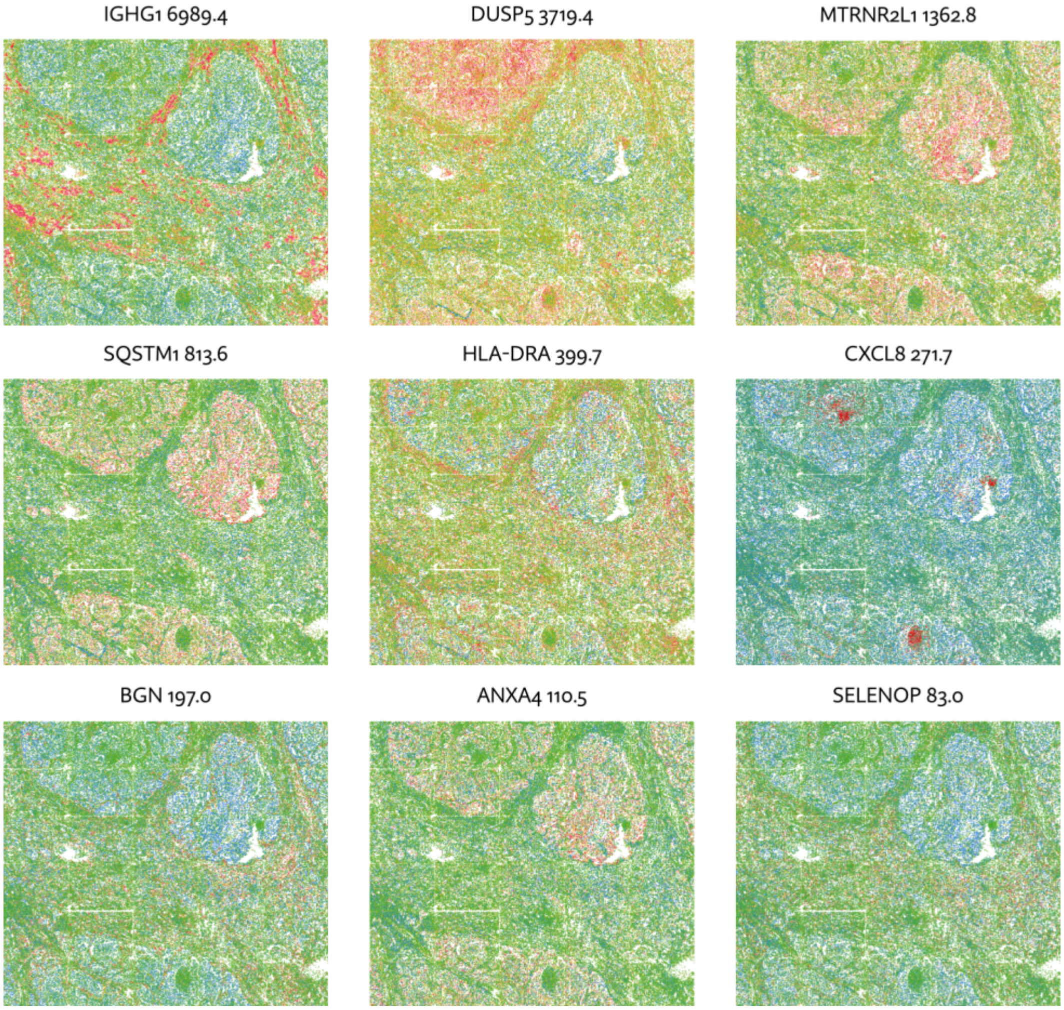

Figure S4: **Examples of genes with corresponding spatial information scores from the renal cell carcinoma CosMx data, related to Figure 4** Genes were selected to show a range of spatial information scores.

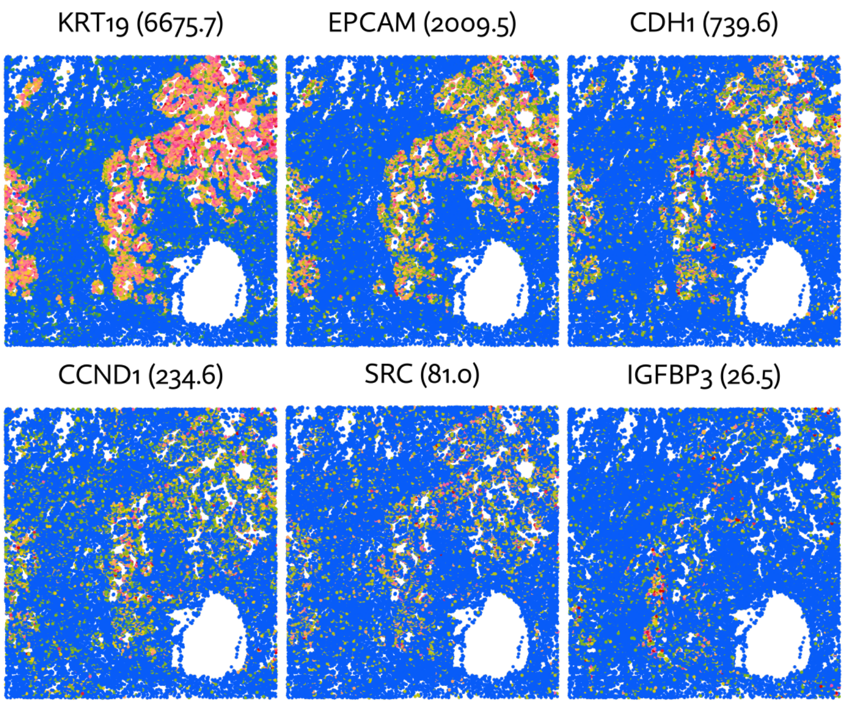

Figure S5: **Examples of genes with corresponding spatial information score, related to Figure 3** Spatial information is seen to strongly correspond to the effect size and the number of cells involved. The highest scoring genes here show elevated expression in the tumor interior.

References

1. Zhang, M., Eichhorn, S. W., Zingg, B., Yao, Z., Cotter, K., Zeng, H., Dong, H., & Zhuang, X. (2021). Spatially resolved cell atlas of the mouse primary motor cortex by MERFISH. *Nature*, *598*, 137-143.
